# Blockade of NMT1 enzymatic activity inhibits N-myristoylation of VILIP3 protein and suppresses liver cancer progression

**DOI:** 10.1038/s41392-022-01248-9

**Published:** 2023-01-09

**Authors:** Xiang-Peng Tan, Yan He, Jing Yang, Xian Wei, You-Long Fan, Guo-Geng Zhang, Yi-Dong Zhu, Zheng-Qiu Li, Hua-Xin Liao, Da-Jiang Qin, Xin-Yuan Guan, Bin Li

**Affiliations:** 1 The First Affiliated Hospital of Jinan University, Jinan University, Guangzhou, China; 2 Key Laboratory of Biological Targeting Diagnosis, Therapy and Rehabilitation of Guangdong Higher Education Institutes and Key Laboratory of Protein Modification and Degradation, The Fifth Affiliated Hospital of Guangzhou Medical University, Guangzhou, China; 3 MOE Key Laboratory of Tumor Molecular Biology and National Engineering Research Center of Genetic Medicine, College of Life Science and Technology, Jinan University, Guangzhou, China; 4 School of Pharmacy, Jinan University, Guangzhou, China; 5 Department of Clinical Oncology, The University of Hong Kong, Hong Kong, China

**Keywords:** Cancer, Drug discovery, Gastrointestinal cancer, Oncogenes, Tumour biomarkers

## Abstract

Hepatocellular carcinoma (HCC) is one of the most common malignant tumors. Identification of the underlying mechanism of HCC progression and exploration of new therapeutic drugs are urgently needed. Here, a compound library consisting of 419 FDA-approved drugs was taken to screen potential anticancer drugs. A series of functional assays showed that desloratadine, an antiallergic drug, can repress proliferation in HCC cell lines, cell-derived xenograft (CDX), patient-derived organoid (PDO) and patient-derived xenograft (PDX) models. N-myristoyl transferase 1 (NMT1) was identified as a target protein of desloratadine by drug affinity responsive target stability (DARTS) and surface plasmon resonance (SPR) assays. Upregulation of NMT1 expression enhanced but NMT1 knockdown suppressed tumor growth in vitro and in vivo. Metabolic labeling and mass spectrometry analyses revealed that Visinin-like protein 3 (VILIP3) was a new substrate of NMT1 in protein N-myristoylation modification, and high NMT1 or VILIP3 expression was associated with advanced stages and poor survival in HCC. Mechanistically, desloratadine binds to Asn-246 in NMT1 and inhibits its enzymatic activity, disrupting the NMT1-mediated myristoylation of the VILIP3 protein and subsequent NFκB/Bcl-2 signaling. Conclusively, this study demonstrates that desloratadine may be a novel anticancer drug and that NMT1-mediated myristoylation contributes to HCC progression and is a potential biomarker and therapeutic target in HCC.

## Introduction

As the most common type of liver cancer, hepatocellular carcinoma (HCC) has a poor prognosis and is the fourth leading cause of cancer-related death worldwide.^1^ Although surgical resection and transplantation are used to treat early-stage HCC, most patients are diagnosed at the late stages.^2^ Sorafenib and lenvatinib are the first-line drugs for the treatment of liver cancer, and the Food and Drug Administration (FDA) approved regorafenib in 2017 for the treatment of sorafenib-resistant liver cancer patients. Despite improvements in chemotherapy in recent decades, the poor survival of patients is a major problem for current cancer therapy.^3–6^ Although the development of sorafenib and regorafenib, the overall survival rate of HCC remains low due to drug resistance and tumor relapse.^7,8^ Therefore, the identification of potential therapeutic agents with antitumor efficacy and low toxicity for HCC patients is urgently needed.^9^

Drug repurposing is the process of developing new uses beyond the original indication of an old drug.^10,11^ Compared with the development of new drugs for a given indication, the strategy of drug repurposing has various advantages; for example, the failure rate is lower, less time for drug development is needed, and monetary cost is mitigated.^12^ In the present study, based on a small molecule library consisting of 419 FDA-approved drugs, we identified desloratadine as a candidate drug for the therapy of HCC. Desloratadine is an orally active H_1_ receptor antagonist that is often used to treat allergies.^13^ However, its potential role in cancer treatment is unknown.

The mechanisms by which desloratadine exerts its antitumor effects remain to be explored. To this end, integrated drug affinity responsive target stability (DARTS) and mass spectrometry-based proteomics technologies were employed to identify the target proteins. N-myristoyl transferase 1 (NMT1), the major enzyme responsible for N-myristoylation, a crucial irreversible eukaryotic lipid modification, was identified as an important target protein of desloratadine. Previous studies demonstrated that NMT1 was overexpressed in various cancers, and NMT1 expression was positively correlated with poor survival of patients.^14^ However, the role of NMT1 in HCC is unclear to date.

To our knowledge, the importance of protein myristoylation in tumorigenesis was first demonstrated by a study suggesting the essential role of myristoylation of the viral oncogene product pp60v-src in membrane transformation.^15^ Studies have increasingly identified protein myristoylation as a target for anticancer chemotherapeutic drugs.^16,17^ Despite the important role of myristoylation in cancer development, the N-myristoylated substrates remain to be identified. Here, a click chemistry approach was adopted, and visinin-like protein 3 (VILIP3) was identified as a novel substrate of NMT1. VILIP3, a member of the visinin-like superfamily,^18^ was reported to increase the proliferation of glioblastoma via the Wnt signaling pathway.^19^ However, the role of VILIP3 in HCC has not been reported.

Here, we aimed to study the regulation and function of NMT1-mediated myristoylation of VILIP3 and examine the anticancer properties of desloratadine as a single drug or in combination with sorafenib in suppressing the progression of HCC in vitro and in vivo. The pharmacological mechanism by which desloratadine directly binds to the NMT1 protein and disrupts the NMT1/VILIP3/NFκB signaling pathway was investigated. Patient-derived organoid (PDO) and patient-derived xenograft (PDX) models were also used for evaluating the treatment efficiency of desloratadine in a preclinical setting.

## Results

### Desloratadine significantly inhibits the proliferation and growth of HCC cells in vitro and in vivo

**Fig. 1:**
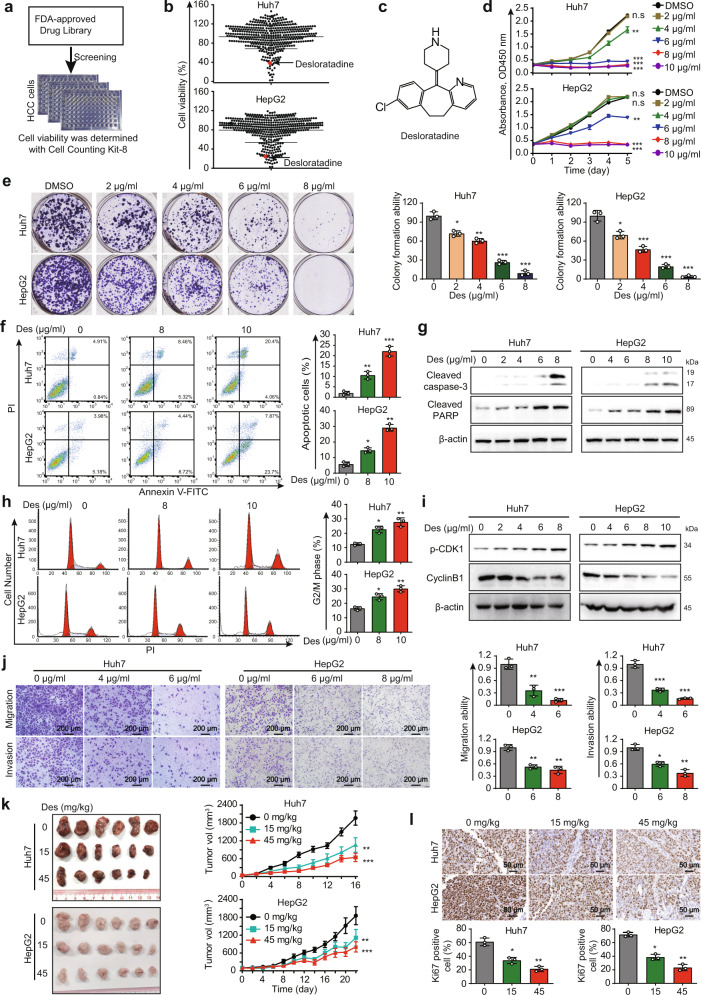
Desloratadine significantly inhibits the proliferation and growth of HCC cells in vitro and in vivo. **a** Diagram showing the procedure for screening an FDA-approved drug library for antitumor drugs. **b** CCK-8 assays showing cell viability upon compound treatment (20 µM, 48 h). **c** Chemical structure of desloratadine. **d** Huh7 and HepG2 cells were treated with different concentrations of desloratadine or vehicle (DMSO), and cell proliferation was evaluated by a CCK-8 assay. **e** The effect of desloratadine on colony formation was monitored. **f** Cells were treated with DMSO or desloratadine for 48 h. The apoptosis rate was analyzed by flow cytometry. **g** Huh7 and HepG2 cells were treated with DMSO or desloratadine for 48 h, and the levels of cleaved PARP and cleaved caspase-3 were determined by Western blot analysis. β-actin served as the internal control. **h** Cells were treated with DMSO or desloratadine for 24 h, and the cell cycle distribution was analyzed by flow cytometry. **i** The level of p-CDK1 and cyclin B1 were analyzed by Western blot. β-actin served as the internal control. **j** Migration and invasion capacities of Huh7 and HepG2 cells with/without desloratadine treatment (4 and 6 µg/ml in Huh7 cells and 6 and 8 µg/ml in HepG2 cells) were compared using Transwell assays. **k** Nude mice bearing Huh7- or HepG2-derived xenografts were orally administered desloratadine (15 mg/kg or 45 mg/kg) or vehicle once every two days (*n* = 6 mice per group). Representative images of the tumors and tumor growth curves showed that desloratadine significantly suppressed the growth of xenograft tumors. **l** Representative images of Ki67 staining in tumor tissue. Bars, SDs; *p < 0.05; **p < 0.01, ***p < 0.001, and n.s., no significance

Based on a drug library consisting of 419 FDA-approved drugs, a drug repurposing approach was applied to identify compounds with anticancer properties in HCC (Fig. 1a and Supplementary Data 1). Firstly, by comparing the suppressive effect of 419 compounds on Huh7 and HepG2 cells, the 20 drugs with inhibitory rate more than 50% were focused (Supplementary Table 1). Secondly, we conducted a literature review for the 20 candidate drugs and found that desloratadine, succinylsulfathiazole and fusidate sodium were rarely reported as anticancer agents, which attracted our attention. Furthermore, our preliminary results showed that desloratadine has a significantly stronger inhibitory effect on HCC cells compared with succinylsulfathiazole and fusidate sodium. (Fig. 1b, c). Therefore, desloratadine became our research focus. The experiments suggested that cell viability was significantly reduced in a dose-dependent manner upon desloratadine treatment (Fig. 1d, e). However, desloratadine did not change the cell viability of MIHA cells (Supplementary Fig. 1a). Furthermore, cell apoptosis was examined in cells treated with desloratadine, and desloratadine induced notable apoptosis of HCC cells compared with that of the control group (Fig. 1f). Consistently, above results were confirmed by the increased cleaved PARP and cleaved caspase-3 expression in the desloratadine-treated HCC cells (Fig. 1g). Integrated cell cycle and Western blot analyses revealed that desloratadine induced G2/M arrest and corresponding changes in the levels of p-CDK1 and cyclin B1 (Fig. 1h, i). Moreover, the migration and invasion of Huh7 and HepG2 cells were significantly impaired by desloratadine, even at a low concentration (Fig. 1j).

In addition, we examined the therapeutic efficacy of desloratadine in vivo. We subcutaneously injecting Huh7 and HepG2 cells into nude mice, and the mice were treated with desloratadine at dosages of 15 or 45 mg/kg once every two days. The results showed that treatment with desloratadine significantly suppressed tumor growth (Fig. 1k), as shown by a decreased Ki67 proliferation index in the desloratadine-treated tumors (Fig. 1l). During the treatment period, body weight was monitored, and no marked difference among the groups was observed (Supplementary Fig. 1b). The serum alanine aminotransferase (ALT) and aspartate aminotransferase (AST) levels also did not significantly change (Supplementary Fig. 1c). Collectively, these results indicate that desloratadine suppresses HCC progression in vitro and in vivo without obvious toxicity.

### High NMT1 expression in HCC is correlated with poor patient survival

**Fig. 2:**
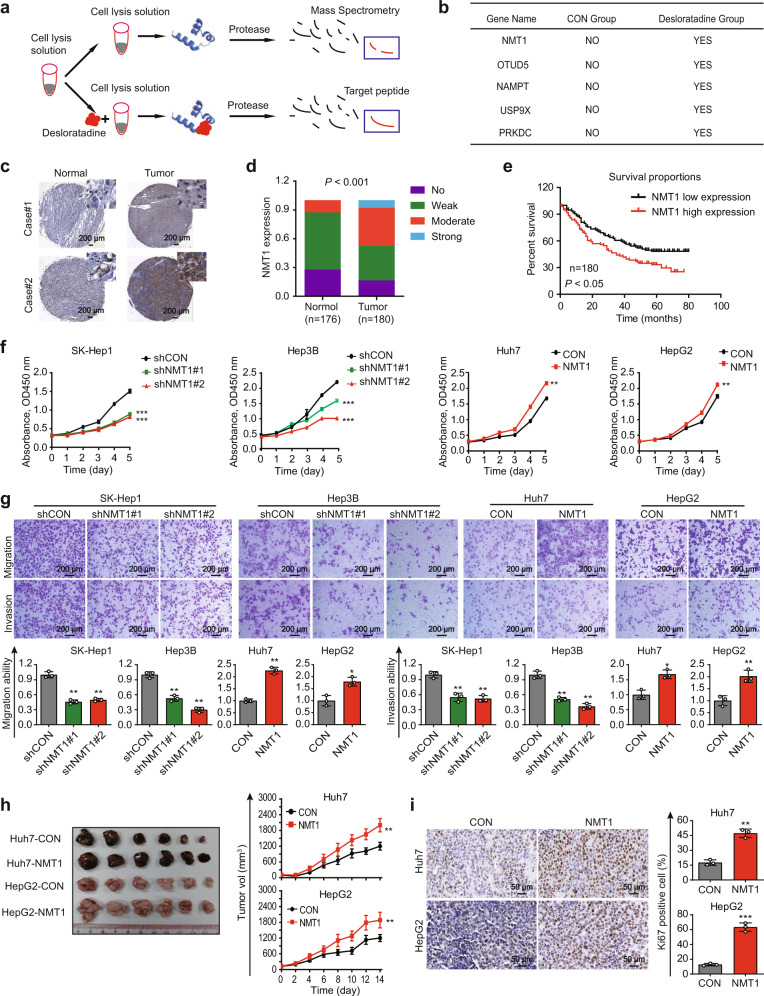
Desloratadine targets the NMT1 protein, which contributes to the progression of HCC. **a** Schematic diagram of the DARTS method. **b** The 5 candidate proteins identified by mass spectrometry analysis. **c** IHC staining showing NMT1 expression in clinical HCC tissues and adjacent noncancerous tissues. **d** NMT1 expression in 180 HCC tumor tissues and 176 matched nontumor tissues. **e** The NMT1 expression level was significantly negatively correlated with the overall survival rate of HCC patients (low NMT1 expression: No and Weak; high NMT1 expression: Moderate and Strong). **f** Cell viability assay showing the effect of NMT1 expression on the viability of HCC cells. **g** Effects of NMT1 expression on the migration and invasion of HCC cells. **h** Image of subcutaneous xenografts derived from nude mice (*n* = 6 tumors per experimental group) and xenograft growth curves. **i** Representative images of Ki67 staining in tumor tissue. Bars, SDs; *p < 0.05; **p < 0.01, ***p < 0.001

To investigate the molecular mechanisms by which desloratadine exerts anticancer bioactivity, the DARTS technology and mass spectrometry analysis were performed (Fig. 2a), and 53 proteins were identified as potential targets of desloratadine (Supplementary Table 2). Among the candidate proteins, 5 protein modification enzymes of interest were selected for further research (Fig. 2b). Notably, knockdown of NMT1, but not OTUD5, USP9X, NAMPT or PRKDC, reduced the sensitivity of HCC cells to desloratadine (Supplementary Fig. 2a, b). NMT2 is another myristoylation enzyme that is highly homologous to NMT1. We didn’t observe the change of NMT1 protein expression when NMT2 was manipulated in Huh7 and HepG2 cells (Supplementary Fig. 2c). More importantly, the data from CCK-8 assay revealed that knockdown of NMT2 in Huh7 and HepG2 cells could not reduce the inhibitory effect of desloratadine on cell proliferation (Supplementary Fig. 2d). Therefore, we selected NMT1 as our research focus. The clinical relevance and functions of NMT1 in HCC remain to be elucidated. NMT1 expression was analyzed in an HCC TMA consisting of 180 tumor tissues and 176 matched nontumor tissues. Significant upregulation of NMT1 was observed in human HCC tissues compared with paired nontumor tissues (Fig. 2c, d and Supplementary Fig. 2e), and high NMT1 expression was significantly associated with advanced pathological T-stage, tumor grade and tumor size (Supplementary Table 3). Moreover, HCC patients with high NMT1 expression had a significantly shorter survival (*p* < 0.05, Fig. 2e). These findings were confirmed by TCGA dataset analysis (Supplementary Fig. 2f, g).

### NMT1 contributes to HCC malignancy

Based on the expression level of NMT1 in HCC cell lines, we overexpressed NMT1 in Huh7 and HepG2 cells and performed NMT1 knockdown in SK-Hep1 and Hep3B cells (Supplementary Fig. 2h, i). And, the protein expression level of NMT2 did not change when we downregulated or upregulated NMT1 expression in HCC cells (Supplementary Fig. 2i). NMT1 downregulation significantly reduced cell proliferation and motility, whereas overexpression of NMT1 exerted the opposite effect (Fig. 2f, g). These findings were confirmed in animal experiments, as indicated by the increased tumor growth and Ki67 proliferation index in xenografts derived from the NMT1-overexpressing Huh7 and HepG2 cells (Fig. 2h, i). Collectively, our data suggest that NMT1 contributes to the tumorigenesis of HCC.

### The binding of desloratadine to Asn-246 of the NMT1 protein is essential for its anticancer bioactivity

**Fig. 3:**
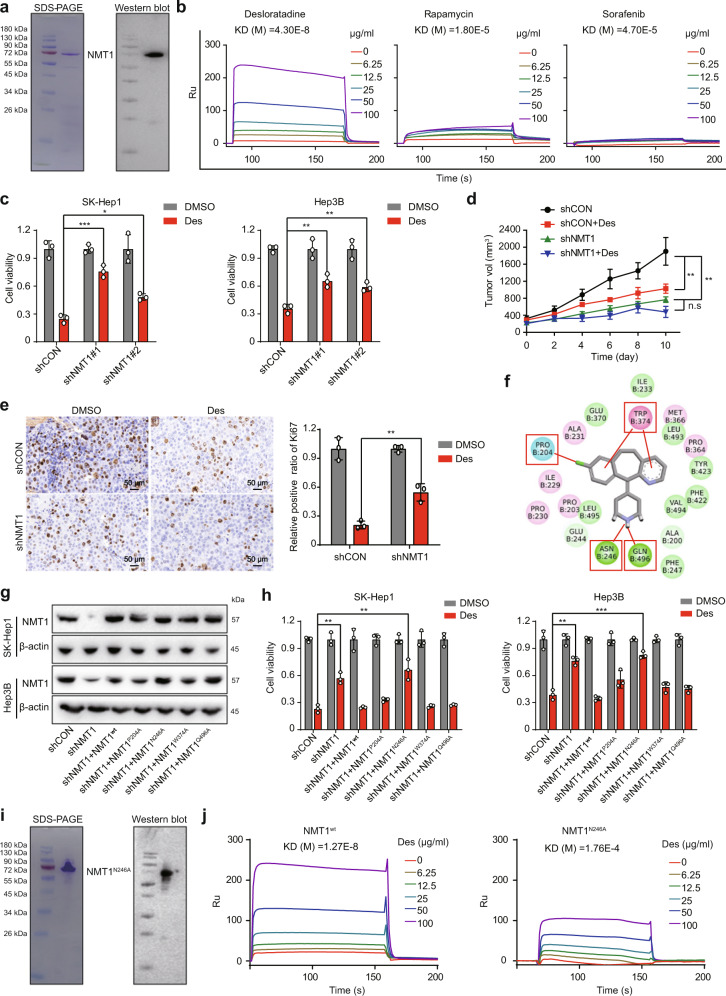
Desloratadine exerts anticancer bioactivity by directly binding to Asn-246 of the NMT1 protein. **a** SDS-PAGE and Western blot analysis indicating the purification of the NMT1 protein. **b** SPR analysis showing the affinity of desloratadine for the NMT1 protein. **c** SK-Hep1-shCON, SK-Hep1-shNMT1, Hep3B-shCON, and Hep3B-shNMT1 cells were treated with 6 µg/ml desloratadine or DMSO, and cell viability was evaluated using a CCK-8 assay. **d** A total of 5 × 10^6^ HCC cells (Hep3B-shCON or Hep3B-shNMT1) were inoculated into nude mice (*n* = mice 6 per group). Tumor-bearing mice were treated with desloratadine (15 mg/kg) or an equal volume of vehicle. **e** Representative images of Ki67 staining in tumor tissue. **f** The binding of NMT1 with desloratadine was investigated by molecular docking simulation. **g** Detection of NMT1 protein expression in each generated cell line by Western blot. **h** Cells expressing mutant or wild-type NMT1 were treated with 6 µg/ml desloratadine or DMSO, and cell viability was evaluated using a CCK-8 assay. **i** Expression and purification of the NMT1^N246A^ mutant protein in a prokaryotic expression system. **j** Detection of the binding of NMT1^N246A^ to desloratadine by SPR analysis, with wild-type NMT1 as the control. Bars, SDs; *p < 0.05; **p < 0.01, ***p < 0.001

Next, we expressed and purified the recombinant NMT1 protein with a prokaryotic expression system (Fig. 3a). As indicated by surface plasmon resonance (SPR) analysis, desloratadine can bind to the NMT1 protein with a high affinity relative to rapamycin and sorafenib as the negative controls (Fig. 3b). More importantly, knockdown of NMT1 substantially decreased the inhibitory effect of desloratadine on cell proliferation (Fig. 3c). Furthermore, in vivo experiments showed that HCC tumor xenografts were less sensitive to desloratadine when NMT1 expression was downregulated (Fig. 3d, e).

To further identify the binding site for desloratadine in NMT1, we conducted molecular docking simulation to predict the interaction of desloratadine with the NMT1 protein. Results indicated that desloratadine may bind to the NMT1 protein by forming hydrogen bonds or van der Waals contacts with the residues proline 204 (P204), asparagine 246 (N246), tryptophan 374 (W374) and glutamine 496 (Q496) in NMT1 (Fig. 3f). Wild-type NMT1 or a series of NMT1 mutants (NMT1^P204A^, NMT1^N246A^, NMT1^W374A^, and NMT1^Q496A^) were re-overexpressed in the NMT1 knockdown cells (Fig. 3g). Interestingly, the reduced sensitivity to desloratadine in the NMT1 knockdown HCC cells was significantly restored in the cells with re-overexpression of wild-type NMT1 or other mutants, but not in the cells with re-overexpression of the NMT1^N246A^ mutant (Fig. 3h). Moreover, we expressed and purified the NMT1^N246A^ protein in a prokaryotic expression system (Fig. 3i). SPR analysis confirmed that the NMT1^N246A^ protein has a lower binding affinity for desloratadine compared with wild-type NMT1 protein (Fig. 3j). Collectively, above results show that desloratadine inhibits the progression of HCC by targeting NMT1 and that Asn-246 in NMT1 is crucial for the binding of desloratadine to the NMT1 protein.

### Desloratadine inhibits NMT1-mediated myristoylation of the VILIP3 protein

**Fig. 4:**
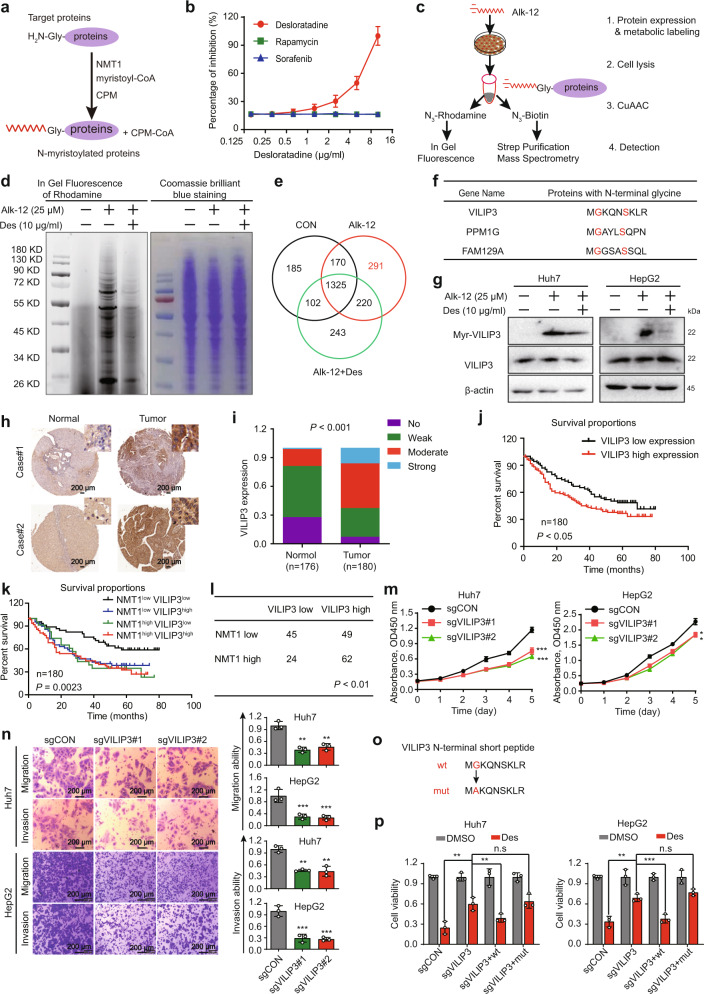
Desloratadine inhibits NMT1-mediated myristoylation of the VILIP3 protein. **a** Schematic diagram showing the approach used to detect NMT1 enzymatic activity. **b** Detection of NMT1 enzymatic activity in the presence of desloratadine, sorafenib or rapamycin. **c** Design of the experiment to identify the substrate protein of NMT1. Alk-12-labeled proteins were conjugated to an azido-rhodamine dye for in-gel visualization or with an azido-biotin reagent for click chemistry, followed by purification and analysis by mass spectrometry. **d** In-gel fluorescence visualization of the myristoylated proteins (left) and the total loaded protein in the NC, Alk-12 and Alk-12+desloratadine groups by Coomassie Brilliant Blue staining (right). **e** Venn diagram of the mass spectrometry results. **f** The potential 3 candidate proteins containing a GxxxS sequence. **g** Western blot showing the level of myristoylated VILIP3 in the presence and absence of desloratadine. **h** IHC staining showing VILIP3 expression in clinical HCC tissues and adjacent noncancerous tissues. **i** VILIP3 expression in 180 HCC tumor tissues and 176 matched nontumor tissues. **j** The VILIP3 expression level was significantly negatively correlated with the overall survival rate of HCC patients (low VILIP3 expression: No and Weak; VILIP3 High expression: Moderate and Strong). **k** Kaplan–Meier analysis of the correlation between the expression of NMT1/VILIP3 and the overall survival of liver cancer patients. We divided patients into the following four groups, including NMT1^low^ VILIP3^low^ group, NMT1^low^ VILIP3^high^ group, NMT1^high^ VILIP3^low^ group and NMT1 ^high^ VILIP3^high^ group. **l** The expression correlation of NMT1 and VILIP3. **m** Cell proliferation was evaluated by a CCK-8 assay in the Huh7 and HepG2 cells transduced with sgCON, sgVILIP3#1 or sgVILIP3#2. **n** The migration and invasion abilities of Huh7 and HepG2 cells expressing sgCON, sgVILIP3#1, or sgVILIP3#2 were evaluated. **o** Schematic diagram of the mutation site in the VILIP3 protein. **p** A series of generated Huh7 and HepG2 cell lines were treated with 6 µg/ml desloratadine or DMSO, and cell viability was evaluated using a CCK-8 assay. Bars, SDs; **p < 0.01, ***p < 0.001

Since both the mRNA and protein levels of NMT1 remained substantially unchanged when HCC cells were exposed to different concentrations of desloratadine (Supplementary Fig. 3a, b), we next determined whether desloratadine affects the enzymatic activity of NMT1. We observed that desloratadine markedly inhibited the enzymatic activity of NMT1, with rapamycin and sorafenib used as negative controls (Fig. 4a, b). We speculated that desloratadine may inhibit the myristoylation of some key proteins by inhibiting the enzymatic activity of NMT1, thereby exerting an antitumor effect. Therefore, a metabolic labeling assay was used to identify the proteins that were not only myristoylated by NMT1 but were also significantly downregulated by desloratadine (Fig. 4c). Rhodamine fluorescence and Coomassie Brilliant Blue staining confirmed that desloratadine significantly decreased the myristoylation level of intracellular proteins in HCC cells (Fig. 4d). Mass spectrometry analysis identified 291 proteins present in the alkynyl myristic acid (Alk-12) group but not in the control group or the desloratadine-treated Alk-12 group, among which 21 proteins were found to have an N-terminal glycine (G) residue (Fig. 4e and Supplementary Table 4). Since the NMT1 protein shows stronger recognition of proteins with an N-terminal amino acid sequence of GxxxS,^20^ we selected three proteins according to these criteria, VILIP3, PPM1G, and FAM129A, for further verification experiments (Fig. 4f). Metabolic labeling assays and Western blot data showed that desloratadine showed stronger inhibition of the N-myristoylation level of the VILIP3 protein than the other 2 proteins (Fig. 4g and Supplementary Fig. 4).

The clinical relevance of VILIP3 remains unclear in HCC. Next, we analyzed a TMA consisting of 180 tumor tissues and 176 matched nontumor tissues and observed that VILIP3 was highly expressed in the tumor tissues than the paired nontumor tissues (Fig. 4h, i and Supplementary Fig. 5a). In particular, VILIP3 level was significantly correlated with pathological stage, tumor grade and patient overall survival (Fig. 4j and Supplementary Table 5). These results were confirmed by TCGA dataset analysis (Supplementary Fig. 5b, c). In addition, we integrated the data of NMT1 and VILIP3 expression from Figs. 2e and 4j, and found that HCC patients with simultaneously higher expression of NMT1 and VILIP3 had poorer overall survival than patients with lower expression of both (Fig. 4k). More importantly, we found that the expression of VILIP3 was positively correlated with NMT1 level in tumor tissues (Fig. 4l). And we performed immunofluorescence to detect NMT1 and VILIP3, our results revealed the co-localization of NMT1 and VILIP3 in HCC cells. (Supplementary Fig. 5d).

To study the biological significance of VILIP3 in HCC, VILIP3-deficient Huh7 and HepG2 cells were generated by the CRISPR/Cas9 system with two individual single-guide RNAs (sgRNAs). We observed significantly decreased cell proliferation, migration and invasion in VILIP3-knockdown cells (Fig. 4m, n and Supplementary Fig. 5e). Thus, we hypothesized that desloratadine inhibits HCC progression by inhibiting NMT1-mediated N-myristoylation of the VILIP3 protein. A VILIP3^mut^ plasmid with mutation of the N-terminal glycine to alanine was constructed (Fig. 4o), and VILIP3-knockout cells were re-overexpressed with VILIP3^wt^ or VILIP3^mut^. Compared with control cells, knockout of VILIP3 significantly reduced the sensitivity of HCC cells to desloratadine, and this effect was abolished by forced expression of VILIP3^wt^ but not VILIP3^mut^ (Fig. 4p and Supplementary Fig. 5f). These data illustrate that NMT1-mediated N-myristoylation of VILIP3 is necessary for the anticancer bioactivity of desloratadine in HCC.

### Desloratadine facilitates the protein degradation of VILIP3 to inhibit the NFκB signaling pathway

**Fig. 5:**
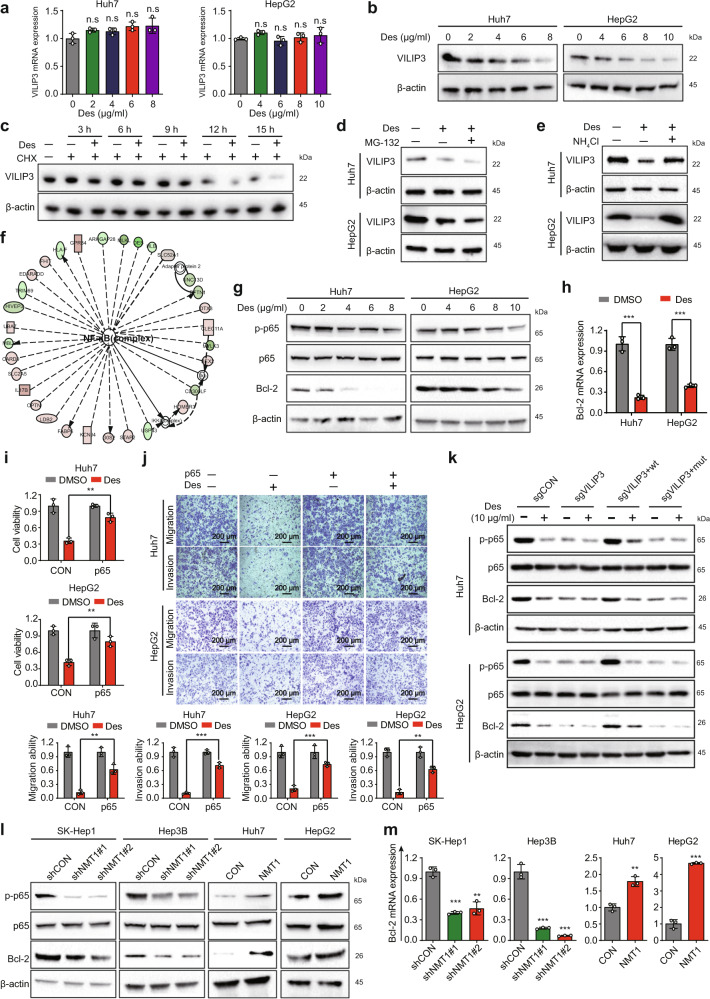
Desloratadine facilitates the protein degradation of VILIP3. **a**, **b** mRNA (**a**) and protein (**b**) expression of VILIP3 was detected in the Huh7 and HepG2 cells treated with different concentrations of desloratadine for 48 h. **c** VILIP3 protein expression in the HepG2 cells treated with CHX alone or in combination with 10 µg/ml desloratadine for different times. **d** VILIP3 protein expression in the Huh7 and HepG2 cells treated with MG-132 (10 µM) alone or in combination with 10 µg/ml desloratadine for 12 h. **e** VILIP3 protein expression in the Huh7 and HepG2 cells treated with NH_4_Cl alone or in combination with 10 µg/ml desloratadine for 12 h. **f** Huh7 cells were treated with DMSO or 10 µg/ml desloratadine for 48 h, RNA sequencing was performed, and IPA identified a significantly altered functional network. **g** Western blot showing the expression of NFκB pathway-related proteins, including p-p65, p65, and Bcl-2. **h** Analysis of the mRNA expression level of Bcl-2, which is a downstream target gene in the NFκB pathway. **i** Huh7-CON, Huh7-p65, HepG2-CON, and HepG2-p65 cells were treated with 6 µg/ml desloratadine or DMSO, and cell viability was evaluated using a CCK-8 assay. **j** Effects of p65 overexpression on desloratadine-inhibited HCC cell migration and invasion. **k** A series of cell lines (sgCON, sgVILIP3, sgVILIP3+wt, sgVILIP3+mut) established from Huh7 and HepG2 cells were treated with desloratadine, and then the expression of p-p65, p65, Bcl-2, and β-actin proteins were detected by Western blot. **l** Western blot detected the expression of the p-p65, p65, Bcl-2, and β-actin proteins in the NMT1 knockdown and NMT1-overexpressing HCC cell lines. **m** The mRNA expression level of Bcl-2 was detected by qRT-PCR. SK-Hep1-shCON, SK-Hep1-shNMT1, Hep3B-shCON, and Hep3B-shNMT1 cells were treated with 10 µg/ml desloratadine or DMSO. Bars, SDs; *p < 0.05; **p < 0.01, ***p < 0.001, and n.s., no significance

To pinpoint the detailed mechanism underlying the regulation of VILIP3 by desloratadine, we measured the RNA and protein levels of VILIP3 in the HCC cells treated with desloratadine. Treatment of HCC cells with desloratadine did not result in an obvious change in the mRNA level (Fig. 5a) but decreased the protein expression of VILIP3 (Fig. 5b). Further cycloheximide (CHX) chase assays showed that desloratadine significantly enhanced the degradation of the VILIP3 protein (Fig. 5c). However, MG-132 (a proteasome inhibitor) did not significantly change the expression level of VILIP3 in the desloratadine-treated HCC cells, indicating that the VILIP3 protein may not be degraded via the ubiquitin-proteasome system (Fig. 5d). Notably, the lysosomal inhibitor ammonium chloride (NH_4_CL) markedly abrogated the protein degradation of VILIP3 induced by desloratadine in HCC cells, suggesting that lysosomal degradation may account for the effect of desloratadine on VILIP3 (Fig. 5e).

To further explore the mechanism by which VILIP3 mediates the anticancer effect of desloratadine, we performed RNA sequencing to identify the differentially expressed genes in the desloratadine-treated HCC cells. Ingenuity pathway analysis (IPA) suggested that the NFκB, Akt/mTOR, ERK1/2, JNK and p38 signaling pathways may play important roles in the anticancer effect of desloratadine (Fig. 5f and Supplementary Fig. 6a). By checking the protein expression, we noted that the NFκB signaling pathway, but not Akt/mTOR, ERK1/2, JNK or p38 signaling pathways, was significantly inhibited by desloratadine in HCC cells (Fig. 5g and Supplementary Fig. 6b). Moreover, desloratadine markedly reduced the mRNA and protein expression of Bcl-2 (Fig. 5g, h), which is not only a critical downstream target of the NFκB pathway but is also closely related to the progression of HCC.^21^ Overexpression of p65 significantly reduced the inhibition of the proliferation, migration, and invasion by desloratadine in HCC cells (Fig. 5i, j and Supplementary Fig. 6c).

To verify the role of VILIP3 in the NFκB signaling pathway, VILIP3-deficient cells were re-expressed with VILIP3^wt^ or VILIP3^mut^, and the cell lines were designated as sgCON, sgVILIP3, sgVILIP3+wt, and sgVILIP3+mut, respectively. Cells were exposed to desloratadine, and then the expression levels of p-p65, p65, Bcl-2, and β-actin were detected. Compared with control cells, the inhibitory effect of desloratadine on NFκB pathway was significantly abolished in VILIP3-deficienct cells, and this effect was restored by overexpression of wild-type VILIP3 but not mutant VILIP3 (Fig. 5k). In addition, NMT1 downregulation reduced but NMT1 upregulation increased the expression levels of phosphorylated p65 (p-p65) and Bcl-2 in HCC cells (Fig. 5l). Consistent results were obtained for the mRNA expression level of Bcl-2 (Fig. 5m). Desloratadine did not markedly alter the expression of p-p65 and Bcl-2 when the NMT1 protein expression was downregulated in HCC cells (Supplementary Fig. 6d). Furthermore, inhibition of the VILIP3/NFκB/Bcl-2 regulatory axis was also observed in the desloratadine-treated Huh7 and HepG2 tumor xenografts (Supplementary Fig. 7). We also analyzed the correlation between NMT1 and 102 downstream genes of NFκB signaling pathway based on the TCGA database. As shown in Supplementary Fig. 6e, NMT1 expression was found to be positively correlated with the NFκB signaling activity in HCC patients. Collectively, our results demonstrate that the downregulation of NMT1 mediates the anticancer effect of desloratadine through inhibition of VILIP3 protein stability and the NFκB/Bcl-2 signaling pathway.

### Desloratadine sensitizes HCC cells to sorafenib treatment

We next investigated whether desloratadine can sensitize HCC cells to sorafenib treatment. We treated HCC cells with desloratadine and sorafenib separately or in combination. Cell viability and colony formation assays showed that although low doses of desloratadine and sorafenib alone had no or only a modest antiproliferative effect, in combination, desloratadine and sorafenib synergistically suppressed cell proliferation (Supplementary Fig. 8a, b). Moreover, data from flow cytometric analysis demonstrated that the combination of desloratadine and sorafenib led to a sharper increase in apoptosis than either agent alone (Supplementary Fig. 8c). Consistent with this finding, the Western blot results showed a significantly increased level of cleaved-PARP in the HCC cells treated with the combination of the two drugs compared with the cells exposed to desloratadine or sorafenib alone (Supplementary Fig. 8d).

To further measure the antitumor effect of desloratadine, we established xenograft models by subcutaneously injecting Huh7 and HepG2 cells into nude mice. Treating the mice with a low dose of desloratadine and sorafenib alone had only a modest antitumor effect; in combination, desloratadine and sorafenib exerted a significant synergistic inhibitory effect on HCC tumor growth (Supplementary Fig. 8e). Histological analysis of critical organs revealed no significant differences among the groups (Supplementary Fig. 8f). In addition, overexpression of NMT1 or VILIP3 significantly reduced the sensitivity of HCC cells to sorafenib (Supplementary Fig. 8g). These results confirm that combination treatment with desloratadine and sorafenib has synergistic effects against HCC cells and tumors.

### Desloratadine suppresses HCC tumorigenesis in PDO and PDX models

**Fig. 6:**
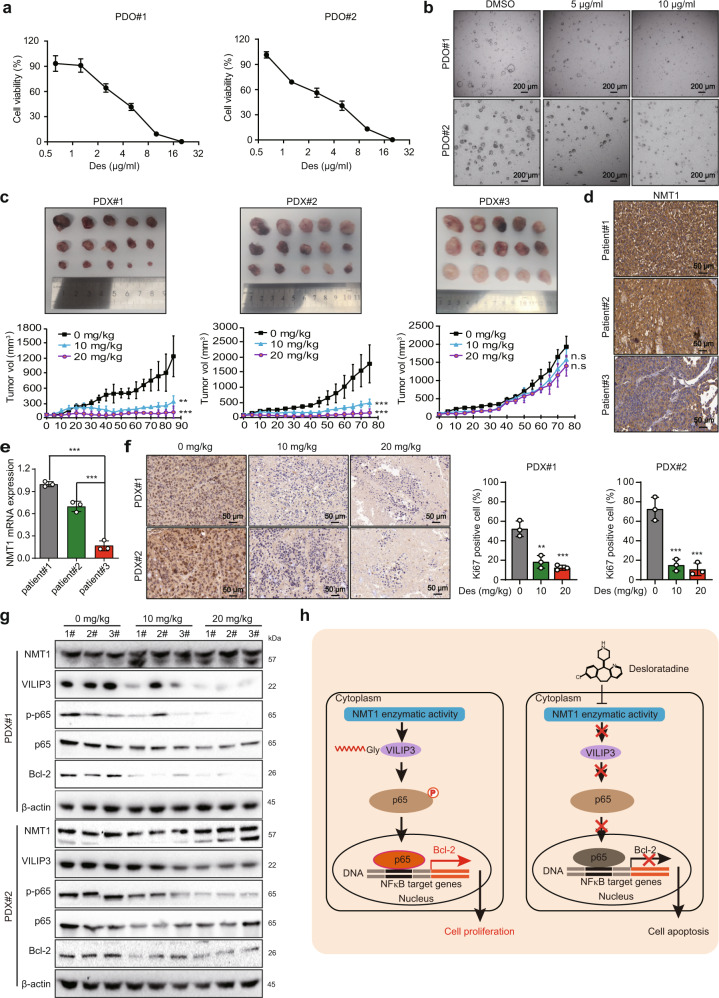
Desloratadine suppresses HCC tumorigenesis in PDO and PDX models. **a** Organoids (PDO#1 and PDO#2) were treated with different concentrations of desloratadine or vehicle (DMSO) for 96 h, and cell viability was evaluated by a CellTiter-Glo Luminescent Cell Viability Assay. **b** The effect of desloratadine on organoid morphology was monitored. **c** Representative images showing the tumor growth curves of mice bearing established PDXs. Mice were fed 100 µl of desloratadine suspension (10 mg/kg or 20 mg/kg body weight) once every five days or an equal volume of vehicle (*n* = 5 per group). **d** Detection of NMT1 protein expression in HCC tissues from three patients by immunohistochemistry. **e** Analysis of the mRNA expression level of NMT1 in HCC tissues from three patients by qRT-PCR. **f** Ki67 staining in tumor tissue sections. **g** Western blot analysis showing the expression of NMT1, VILIP3, and NFκB signaling pathway-related proteins in the xenograft tumors. **h** Schematic diagram summarizing how desloratadine suppresses NMT1 enzymatic activity and tumorigenesis. Bars, SDs; *p < 0.05; **p < 0.01, ***p < 0.001, and n.s., no significance

PDO and PDX models have become important preclinical model systems for evaluating drug responses and drug efficacy. First, the tumor tissues from 2 HCC patients were obtained to construct PDO models. We found that treatment with desloratadine markedly inhibited the viability of PDO#1 and PDO#2 cells in a dose-dependent manner (Fig. 6a, b). The Hoechst 33342 and propidium iodide (PI) staining analysis showed that desloratadine significantly increased apoptosis in PDO#1 and PDO#2 cells (Supplementary Fig. 9a).

In PDX models, treatment with desloratadine significantly suppressed the growth of PDX#1 and PDX#2, but not PDX#3, in a dose-dependent manner (Fig. 6c). Notably, both the mRNA and protein expression level of NMT1 in the tumor tissues of patient #1 and patient #2 was higher than that in patient #3 (Fig. 6d, e). Moreover, IHC and terminal deoxynucleotidyl transferase (TDT)-mediated dUTP-fluorescein nick end labeling (TUNEL) assays showed that desloratadine significantly induced apoptosis and reduced the Ki67 proliferation index in PDX#1 and PDX#2 (Fig. 6f and Supplementary Fig. 9b). Furthermore, inhibition of the NMT1/VILIP3/NFκB/Bcl-2 regulatory axis was observed in the desloratadine-treated PDX#1 and PDX#2 tumor xenografts, as indicated by Western blot (Fig. 6g). Histological analysis of critical organs revealed no significant differences among the groups, further suggesting the safety of desloratadine (Supplementary Fig. 9c). Collectively, these results suggest the potential of desloratadine as a precision medicine tailored for patients with high NMT1 expression.

## Discussion

Myristoylation refers to the covalent binding of myristic acid to a glycine residue at the N-terminus of a protein.^22–24^ Nearly 2% of the proteins in cells are reported to exhibit N-myristoylation,^25^ which can affect protein interaction and stability.^26,27^ The dysregulation of protein myristoylation is strongly linked to various cancers.^28^ Inhibition of the myristoylation modification of Src protein was reported to block its kinase activity and inhibit the progression of prostate cancer.^29^ As one of the important enzyme, NMT1 can recognize and catalyze the myristoylation of proteins with a GxxxS sequence at the N-terminus.^[20,30^ However, studies on the catalytic substrate proteins of NMT1 are limited. In this study, by integrating a click chemistry and mass spectrometry approach, we identified VILIP3 as a new substrate protein of NMT1. The biological role of VILIP3 in cancer is controversial. VILIP3 was shown to suppress cancer progression by promoting p21 stabilization,^31^ whereas other studies reported that VILIP3 promotes glioblastoma cell proliferation.^19^ Here, we provide the first evidence showing that the VILIP3 protein is a new catalytic substrate protein of NMT1 and that myristoylation of VILIP3 affects its stability to activate the NFκB/Bcl-2 regulatory axis. VILIP3 overexpression in HCC patients was correlated with poor survival, and more importantly, knockdown of VILIP3 was found to inhibit cell proliferation and motility, suggesting that VILIP3 may be a useful biomarker and therapeutic target in HCC.

Accumulating evidence has revealed that NMT1 is highly expressed in various cancers,^32–34^ and high expression of NMT1 is closely related to poor prognosis in cancer patients. For example, NMT1 was reported to promote the tumor progression by inhibiting autophagy through the endoplasmic reticulum (ER) stress pathway in breast cancer.^14^ However, the role of NMT1 in HCC remains unclear, and its molecular mechanism in the progression of HCC has not been elucidated. Here, gain- and loss-of-experiments and a series of in vitro and in vivo assays illustrated that NMT1 overexpression contributes to HCC cancer progression through the myristoylation of downstream substrates and the activation of key signaling pathways. The IHC analysis showed that NMT1 upregulation is frequently observed in HCC and related to poor prognosis. The correlation between expression of NMT1 and the sensitivity of sorafenib in HCC patients and whether NMT1 could be developed as a therapeutic target for the treatment of HCC warrants further investigation.

As a strategy for drug development, drug repurposing has some unparalleled advantages, such as shorter drug development times, lower risks of failure and lower drug development costs.^12,35^ There are many successful examples of drug repurposing, such as thalidomide and disulfiram.^36–38^ Desloratadine is an orally active H_1_ receptor antagonist that is often used to treat allergies.^13^ Some H_2_ antihistamines have shown promise in cancer therapy, but modern H_1_ antihistamines have not been widely studied in this role.^39^ A third-generation antihistamine, desloratadine, was reported to cause fewer adverse drug reactions in the central nervous system than first- and second-generation antihistamines.^40^ In this study, the direct binding between the NMT1 protein and desloratadine was confirmed by SPR analysis and molecular docking simulation, and Asn-246 in NMT1 was identified to play a crucial role in the binding of NMT1 to desloratadine. We first found that desloratadine can inhibit the proliferation, migration and invasion of HCC cells. In addition, desloratadine markedly increased apoptosis of HCC cells and reduced the volume of tumor xenografts in animal models. Our results showed that desloratadine inhibited the progression of HCC through suppressing the enzyme activity of NMT1. Other factors may be involved in the anticancer effect of desloratadine and the underlying mechanism may be further investigated. More importantly, preclinical studies in PDO and PDX models further confirmed the anticancer of desloratadine in suppressing HCC progression. Evaluation of desloratadine bioactivity in spontaneous tumor models and immunocompetent mouse models could further support the development of desloratadine as an anticancer agent in the clinic.

Chemotherapeutic resistance leads to cancer recurrence and poor clinical outcomes in HCC.^41,42^ Combination treatment with two or more compounds with different mechanisms of action is an alternative approach to increase the success rate of chemotherapy.^43,44^ Our present study indicated that treatment with desloratadine can sensitize HCC cells to traditional chemotherapeutic drugs. In summary, we demonstrated that NMT1 promotes HCC tumorigenesis by enhancing the myristoylation and stability of the VILIP3 protein. Desloratadine directly binds to NMT1 to suppress the progression of HCC by inhibiting the enzymatic activity of NMT1 and subsequent VILIP3/NFκB/Bcl-2 signaling (Fig. 6h). These findings not only highlight the potential of NMT1 as a prognostic biomarker and therapeutic target in HCC but also support the use of desloratadine in cancer treatment.

## Materials and methods

### Cell lines and culture conditions

In this study, an immortalized human hepatocyte cell line (MIHA) and human HCC cell lines (Huh7, HepG2, SK-Hep1, and Hep3B) were obtained from the Cell Bank of the Chinese Academy of Sciences (Shanghai, China), and the 293T cell line was purchased from the American Type Culture Collection (ATCC) (Rockville, MD, USA) and cultured according to the instructions. Cells were confirmed to have no mycoplasma contamination and were passaged no more than 25 to 30 times after thawing.

### Plasmids, transfection, and site-directed mutagenesis

The plasmids expressing human NMT1 and VILIP3, as well as the NMT1 knockdown plasmids, were obtained from TranSheepBio (Shanghai, China). The small guide RNA (sgRNA) sequences targeting VILIP3 were cloned into the LentiCRISPRv2 plasmid. The procedures for virus packaging, cell transduction and cell selection were performed as described previously.^45^ Cells were transfected with small interfering RNA (siRNA) (TranSheepBio) by using Lipofectamine 3000 according to the manufacturer’s instructions. The sequences of short hairpin RNA (shRNA), sgRNA, and siRNA are listed in Supplementary Table 6.

### Western blot analysis

Cells were lysed with RIPA lysis buffer (Cell Signaling Technology, Beverly, MA, USA) according to the manufacturer’s instructions. Protein concentration was determined with a BCA kit (Thermo Fisher Scientific, Waltham, MA, USA). The next steps in the experimental process were carried out as previously described.^46^ Samples were loaded on SDS-PAGE gel and electrotransferred onto PVDF membrane (Millipore, Bedford, MA, USA). The membrane was blocked and then incubated with a primary antibody at 4 °C overnight. After the membrane was washed, it was incubated with an appropriate secondary antibody (Cell Signaling Technology). Protein bands were visualized using ECL reagents (Bio-Rad, Hercules, CA, USA). The antibodies used in this study are listed in Supplementary Table 7.

### qRT-PCR assay

Total RNA was extracted with a HiPure Total RNA Mini Kit (Magen, Guangzhou, China). cDNA was synthesized with TransScript One-Step gDNA Removal and cDNA Synthesis SuperMix (TransGen, Beijing, China), and qRT-PCR was then performed in a Bio-Rad Mini Opticon real-time PCR system using iTaqTM Universal SYBR Green Supermix (Bio-Rad) according to the manufacturer’s instructions. GAPDH was used as the internal control. The primer sequences specific for each gene are listed in Supplementary Table 8.

### Cell viability assay

Cell viability was evaluated with a Cell Counting Kit-8 (CCK-8, Selleck Chemicals, Houston, Texas, USA) according to the instructions. In brief, hepatocellular carcinoma (HCC) cells were cultured at 1000 cells per well in 96-well plates and treated with different concentrations of desloratadine. The absorbance was measured at 450 nm after treatment with 10 μl of CCK-8 reagent (Selleck Chemicals) for 2 h.

### Colony formation assay

The assay was performed as described previously.^47^ HCC cells were treated with drugs for 10–14 days. Colonies were fixed and then stained with crystal violet. Finally, the colony numbers were counted.

### Migration and invasion assays

Migration and invasion assays were performed as described previously.^47^ In migration assay, cells in serum-free medium were added to the upper chamber (Corning, New York, USA), and complete medium was added to the bottom wells of chamber as a chemoattractant. The migrated cells were counted after 12 h. Images were acquired in three different fields of view. For invasion assay, same methods were taken to detect the invasive ability of cells in chambers with a Matrigel (Corning)-coated membrane.

### Cell cycle analysis

Cell cycle assay was conducted as described in a previous study.^48^ Cells in different groups were harvested, washed with PBS and fixed with 75% ethanol for 2 h. After staining with PI/RNase solution for 15 min in the dark, cell-cycle profiles were determined using flow cytometry (BD Biosciences, Bedford, MA, USA).

### Apoptosis assay

An Annexin V-FITC/PI Apoptosis Detection Kit (KeyGen, Nanjing, China) was used to evaluate apoptosis.^49^ Cells were incubated with 5 μl of FITC-conjugated Annexin V and 5 μl of PI for 15 min at room temperature in the dark, and 400 μl of binding buffer was then added. Apoptotic cells were analyzed in a FACSCalibur flow cytometer (BD Biosciences).

### TUNEL assay

In brief, after deparaffinization and antigen retrieval, tumor tissue sections were permeabilized with fresh working solution (Servicebio, Wuhan, China). Next, appropriate amounts of TDT enzyme and the dUTP mixture were added to the sections at 37 °C. After three washes, the sections were stained with DAPI (Servicebio) and coverslipped with antifade mounting medium (Servicebio). Finally, the stained sections were imaged by fluorescence microscopy (Nikon, Tokyo, Japan).

### DARTS analysis

Cell lysates from independent biological replicates were aliquoted in equivalent volumes containing 100 µg of protein and incubated for 10 min at 25 °C with or without desloratadine. Proteinase K from *Tritirachium album* (Sigma-Aldrich, St. Louis, MO, USA) was added simultaneously to all samples at a proteinase K: substrate mass ratio of 1:100 and incubated at 25 °C for 5 min. The detailed steps were described previously.^50^

### Expression and purification of the NMT1 protein

The pET-28a plasmids expressing His-tagged wild-type or mutant NMT1 were transformed into *Escherichia coli* BL21 Star (DE3) cells as previously described.^51^ NMT1 protein expression was induced by 0.1 mM isopropyl β-D-thiogalactopyranoside (IPTG) and incubated for 48 h at 15 °C with shaking at 120 rpm. Next, bacterial cells were lysed by sonication, and the collected supernatant was loaded onto a Ni-NTA Superflow affinity column. Finally, the His-tagged NMT1 fusion protein was eluted with 150 mM imidazole. The eluted protein was analyzed by SDS-PAGE.

### SPR analysis

Binding between inhibitors and NMT1 was assessed by SPR with Biacore X100 instrument (GE Healthcare Life Sciences, Marlborough, MA, USA). A series of analytes (desloratadine, sorafenib and rapamycin; sorafenib and rapamycin were used as the negative controls) were prepared at different concentrations in running buffer [1% DMSO and 0.05% polysorbate 20 (a surfactant)]. NMT1 was the ligand and was coupled to the CM7 sensor chip (GE Healthcare Life Sciences), while the inhibitors were used as the analytes. The detailed experimental procedures were described previously.^51^

### Fluorescence-based N-myristoyltransferase activity assay

Fluorescence-based N-myristoyltransferase activity assay was conducted as previously described.^52^ 10 µl volumes of different dilutions of desloratadine in 10% DMSO/water (v/v), 25 µl of myristoyl–CoA (Sigma-Aldrich) solution (4 µM), 50 µl of NMT1 protein (6.3 nM), and 25 µl of CPM (Abcam, Cambridge, UK) solution (40 µM) were combined in an assay well. The enzymatic reaction was started when 25 µl of peptide (Sangon Biotech, Shanghai, China) substrate solution (20 µM) was added. The reaction was stopped after 30 min at 25 °C by adding 60 µl of quenching solution (0.1 M sodium acetate buffer, pH 4.75). The fluorescence intensity was monitored at 1 min intervals (excitation at 362 nm, emission at 470 nm) at 25 °C.

### Detection of the NMT1 substrate protein by metabolic labeling assay

For evaluation of protein myristoylation, a click chemistry approach was used essentially as described previously.^53^ Briefly, HepG2 cells were cultured for 8 h in Dulbeccoʼs modified Eagle’s medium (DMEM; Invitrogen, Carlsbad, CA) containing 2% fetal bovine serum (FBS; HyClone, UT, USA), alkyl myristic acid (Alk-12; Click Chemistry Tools, New Jersey, USA) or Alk-12 and desloratadine. Proteins were extracted by cell lysis using RIPA buffer, and 2 mg of protein per sample was conjugated to a TAMRA-azido-biotin probe (Click Chemistry Tools). Next, the biotin-labeled proteins were pulled down with streptavidin magnetic beads (Thermo Fisher Scientific). Protein myristoylation was detected by rhodamine fluorescence, and proteins were processed for mass spectrometry.

### RNA sequencing (RNA-seq) and ingenuity pathway analysis (IPA)

Total RNA was extracted from cells using the HiPure Total RNA Mini Kit (Magen). Then, the RNA samples were sent for RNA-seq analysis to the Beijing Genomics Institute Tech Solutions Co. (Shenzhen, China). A difference of ≥2 fold was defined as differential expression. Compared with the DMSO group, the differentially expressed genes were subjected to IPA analysis, and the signaling pathway was analyzed via the IPA software (Ingenuity Systems, Redwood City, CA, USA).

### PDO tumor models

Fresh tumor tissue obtained from liver cancer patients was cut into pieces, washed with PBS, and digested with Accuroid Tissue Dissociation Solution (Accurate International Biotechnology, Guangzhou, China). After 100 μm filtration, centrifugation and erythrocyte lysis, cells were resuspended in Advanced DMEM/F12 medium (Gibco) were mixed with an equal volume of Matrigel, and then, Accuroid Organoid Culture Medium-Liver Cancer (Accurate International Biotechnology) was added to cover the Matrigel and cells were cultured in a CO_2_ cell incubator at 37 °C. The medium was changed every two days, and the organoids were cultured for 7–14 days for subculture and related follow-up experiments.

### Tumor xenograft experiments

The experiment was performed as previously described.^51^ Nude mice were subcutaneously injected with HCC cells (5 × 10^6^). Once the tumor volume reached 100 mm^3^, the mice were treated with the drug every two days. Treatment was continued for 2–3 weeks before sacrifice, and the tumors were subjected to IHC staining and analysis. All animal procedures were conducted following the procedures approved by the Ethics Committee for Animal Experiments of Jinan University. Male BALB/c nude mice aged 6–8 weeks were maintained under standard conditions and cared for according to the institutional guidelines for animal care.

### PDX tumor models

Fresh tumor tissues from liver cancer patients were collected and cut into small pieces of the appropriate size. These tumor pieces were then transplanted into the right subcutaneous area of NOD-Prkdc^scid^-Il2rg^em1IDMO^ mice (Beijing IDMO Co., Ltd., Beijing, China). Mice were sacrificed when the tumor volume was 1000 mm^3^. These tumor tissues were cut into small pieces (2 mm^3^) and were then transplanted into the right subcutaneous area of mice. When the tumor size was ~100 mm^3^, these mice were randomly divided into groups and treated as needed. Xenograft tissue was snap frozen and stored at −80 °C or processed by formalin fixation. Use of all human samples was approved by the Ethics Committee of Jinan University. The informed consents were all obtained.

### Clinical samples and IHC analysis

A tissue microarray (TMA) consisting of 180 paired liver cancer and corresponding adjacent nontumor liver tissues was purchased from Shanghai Outdo Biotech Co., Ltd. (Shanghai, China). IHC analysis was performed as previously described.^54^ After antigen retrieval and blocking, sections were incubated with primary antibodies against NMT1 (Abcam), VILIP3 (Proteintech) or Ki67 (Cell Signaling Technology, Beverly, MA, USA) overnight at 4 °C. After washing with PBS, the sections were incubated with a secondary antibody for 1 h and developed with diaminobenzidine (DAB) (Servicebio). Finally, the sections were stained with hematoxylin and sealed with neutral resin. All stained sections were scanned using fluorescence microscopy (Nikon). The staining score was graded as follows: 0, no staining; 1, low staining; 2, moderate staining; and 4, strong staining. Spots with 0 or 1 scores were regarded as low expression, while 2 or 3 were high expression.

### Statistical analysis

All measurement data are expressed as the mean ± SD values and were analyzed using GraphPad Prism software (San Diego, CA, USA). For comparisons between two groups, Student’s t test was used. For comparisons among more than two groups, one-way analysis of variance (ANOVA) was used. *p < 0.05, **p < 0.01, and ***p < 0.001 were considered significant.

## Supplementary information

Supplementary information

Supplementary Data 1

## Data Availability

All data needed to evaluate the conclusions in the article are present in the article and/or the Supplementary Materials. The data and materials used in the current study are available from the corresponding authors upon reasonable request.
