# Supplementary material for: Blockade of NMT1 enzymatic activity inhibits N-myristoylation of VILIP3 protein and suppresses liver cancer progression
Source: Signal Transduct Target Ther. 2023 Jan 9;8:14. doi: 10.1038/s41392-022-01248-9 (PMC9826789; doi:10.1038/s41392-022-01248-9)
Supplement: Supplementary file 1 — Supplementary information [file 41392_2022_1248_MOESM1_ESM.docx]

Supplementary Materials for

Blockade of NMT1 enzymatic activity inhibits N-myristoylation of VILIP3 protein and suppresses liver cancer progression

Xiang-Peng Tan^1,2#^, Yan He^2,3#^, Jing Yang^2,3^, Xian Wei^2^, You-Long Fan^4^, Guo-Geng Zhang^2,3^, Yi-Dong Zhu^2,3^, Zheng-Qiu Li^4^, Hua-Xin Liao^3^, Da-Jiang Qin^2^, Xin-Yuan Guan^5^, Bin Li^2*^

Correspondence to: lib2128@163.com

**This PDF file includes:**

Figures. S1 to S9

Tables S1 to S8

Captions for Data S1


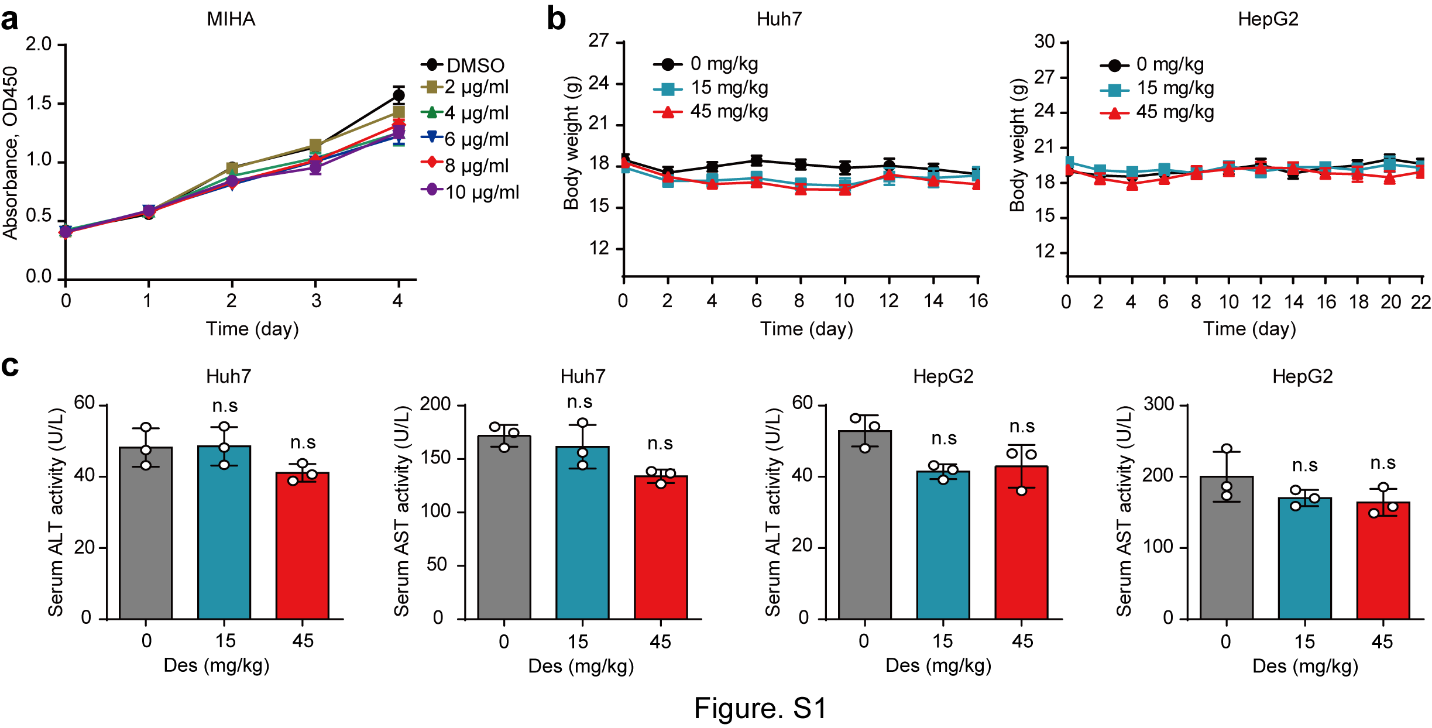


Figure. S1. The toxicity of desloratadine was evaluated. a MIHA cells were treated with different concentrations of desloratadine or vehicle (DMSO), and cell proliferation was evaluated by a CCK-8 assay. b Body weights of mice were monitored upon drug administration. c Serum ALT and AST levels were measured in the indicated groups. Bars, SDs; n.s, no significance.


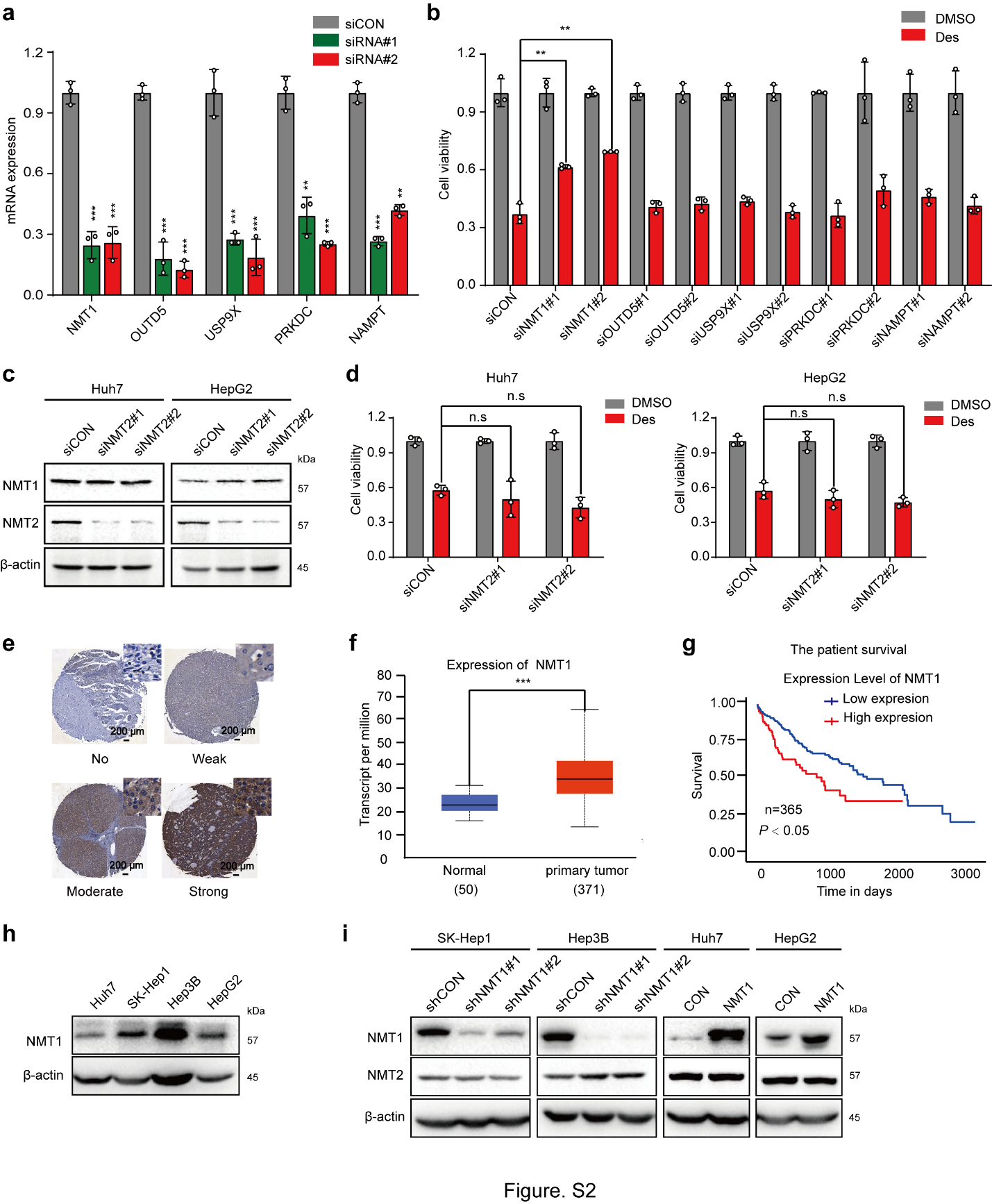


Figure. S2. Identification and characterization of NMT1 as a direct target of desloratadine. a Successful knockdown of the five candidate target proteins of desloratadine was confirmed by qRT-PCR. b Knockdown of NMT1, but not other candidate targets, attenuated the anticancer effect of desloratadine in HepG2 cells. c The protein expression of NMT1 and NMT2 in the NMT2-knockdown cells. d Huh7 and HepG2 cells were transfected with siNMT2 or siCON, and treated with 6 µg/ml desloratadine or DMSO, and cell viability was evaluated using a CCK-8 assay. e NMT1 expression in human HCC tissues and adjacent noncancerous tissues was detected by immunohistochemistry. According to the NMT1 protein expression level, the tissues were classified into four categories: No, Weak, Moderate and Strong. f TCGA database analysis was used to assess the expression pattern of NMT1 in the HCC cohort. g The NMT1 expression level was significantly negatively correlated with the overall survival rate of HCC patients. h NMT1 protein expression in four different HCC cell lines. i Expression of the NMT1 and NMT2 proteins in the NMT1 knockdown and NMT1-overexpressing HCC cell lines. Bars, SDs; **, p <0.01, ***, p <0.001.


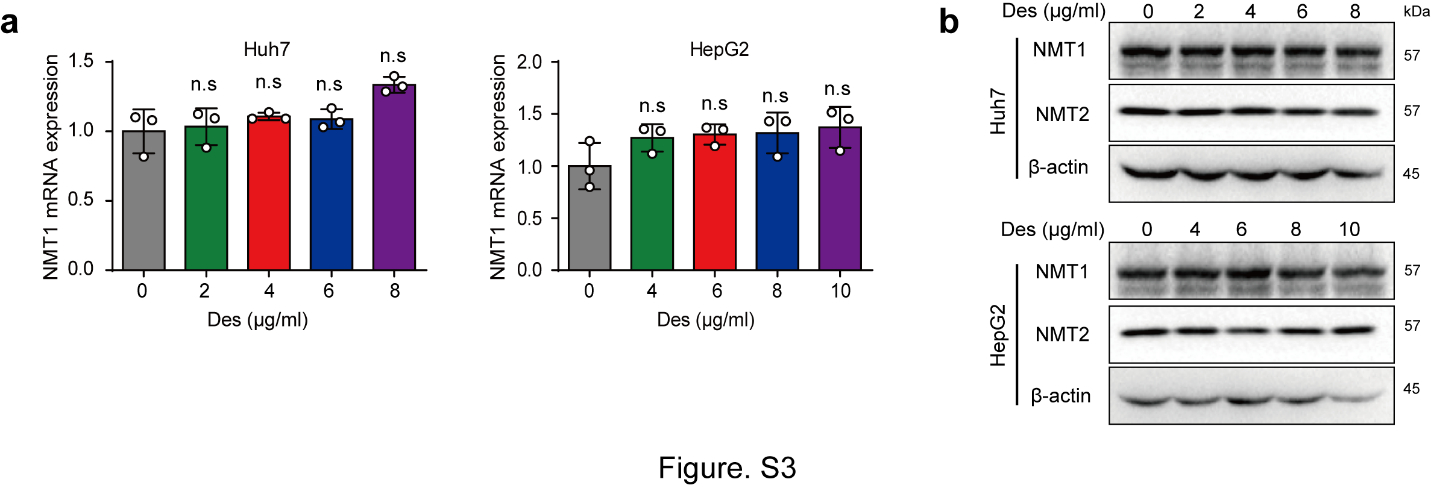


Figure. S3. Effect of desloratadine on the expression of NMT1 in HCC cells. a Detection of the mRNA expression of NMT1 in the Huh7 and HepG2 cells treated with different concentrations of desloratadine for 48 h by qRT-PCR. b Western blot showing the expression of the NMT1 and NMT2 protein in the HCC cells treated with different concentrations of desloratadine for 48 h. Bars, SDs; n.s, no significance.


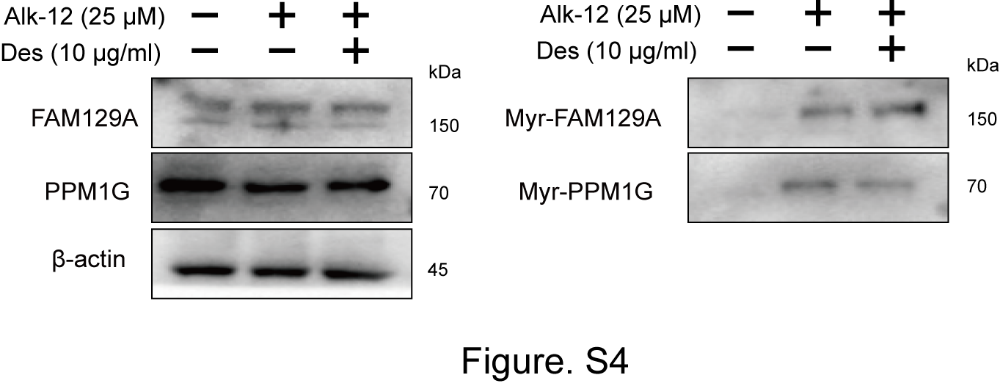


Figure. S4. Effect of desloratadine on FAM129A and PPM1G protein myristoylation.


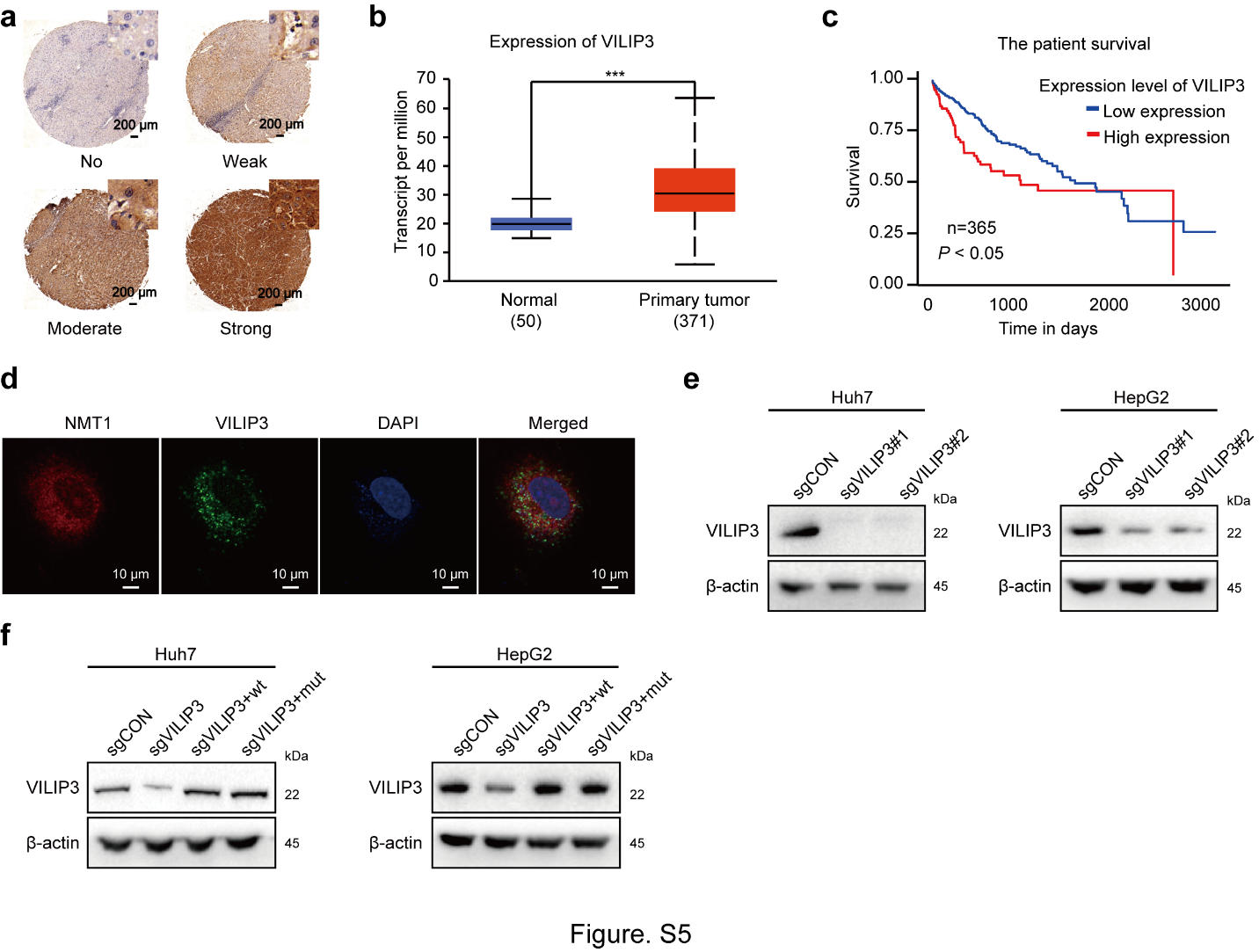


Figure. S5. The expression and clinical significance of VILIP3 in HCC. a VILIP3 expression in clinical HCC tissues and adjacent noncancerous tissues was detected by immunohistochemistry. According to the VILIP3 protein expression level, the tissues were classified into four categories: No, Weak, Moderate and Strong. b TCGA database analysis was used to assess the correlations of VILIP3 expression patterns of in the HCC cohort. c The VILIP3 expression level was significantly negatively correlated with the overall survival rate. d The co-localization of NMT1 and VILIP3 in Huh7 cells. e Expression of the VILIP3 protein in the VILIP3 knockout HCC cells. f Western blot showing the expression of VILIP3 among the groups.


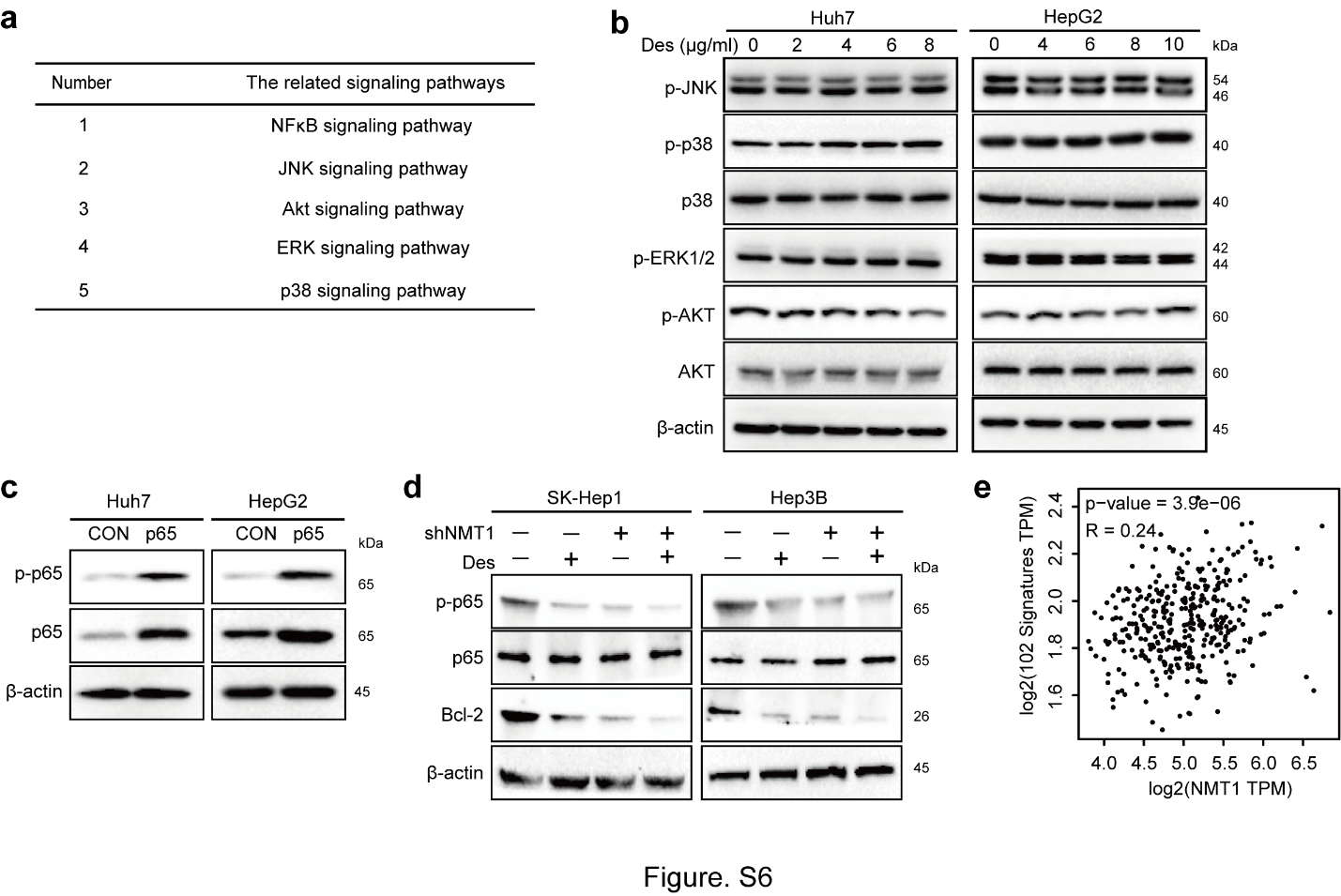


Figure. S6. Effect of desloratadine on the potential downstream signaling pathways in HCC cells. a The list of potential signaling pathways analyzed by IPA. b Huh7 and HepG2 cells were treated with DMSO or desloratadine for 48 h, and the levels of p-JNK, p-p38, p38, p-ERK1/2, p-Akt, Akt and β-actin were determined by Western blot analysis. c Overexpression of p65 in Huh7 and HepG2 cells. d In the presence or absence of desloratadine as indicated, the expression of p-p65, p65 and Bcl-2 was monitored in NMT1-knockdown HCC cells. e The correlation between NMT1 and 102 downstream genes of NFκB signaling pathway by analyzing TCGA database.


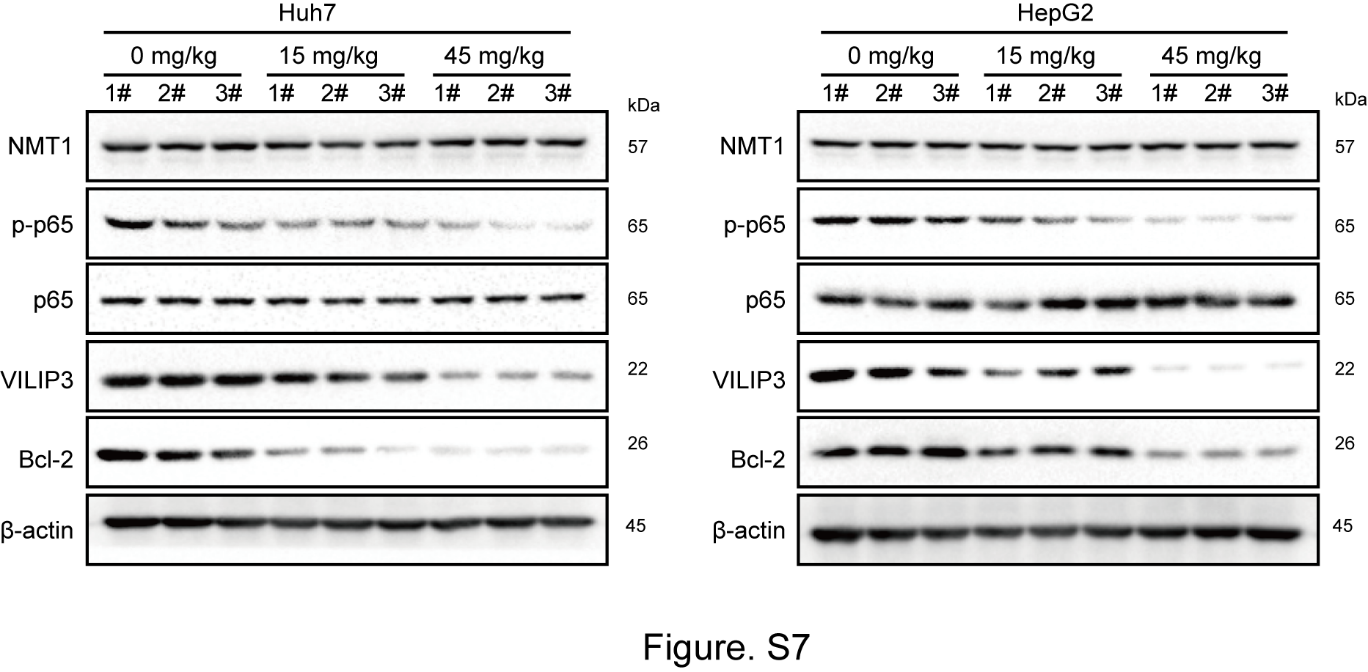


Figure. S7. The expression of NMT1, VILIP3, Bcl-2 and NFκB signaling pathway-related proteins in the desloratadine-treated tumor xenografts.


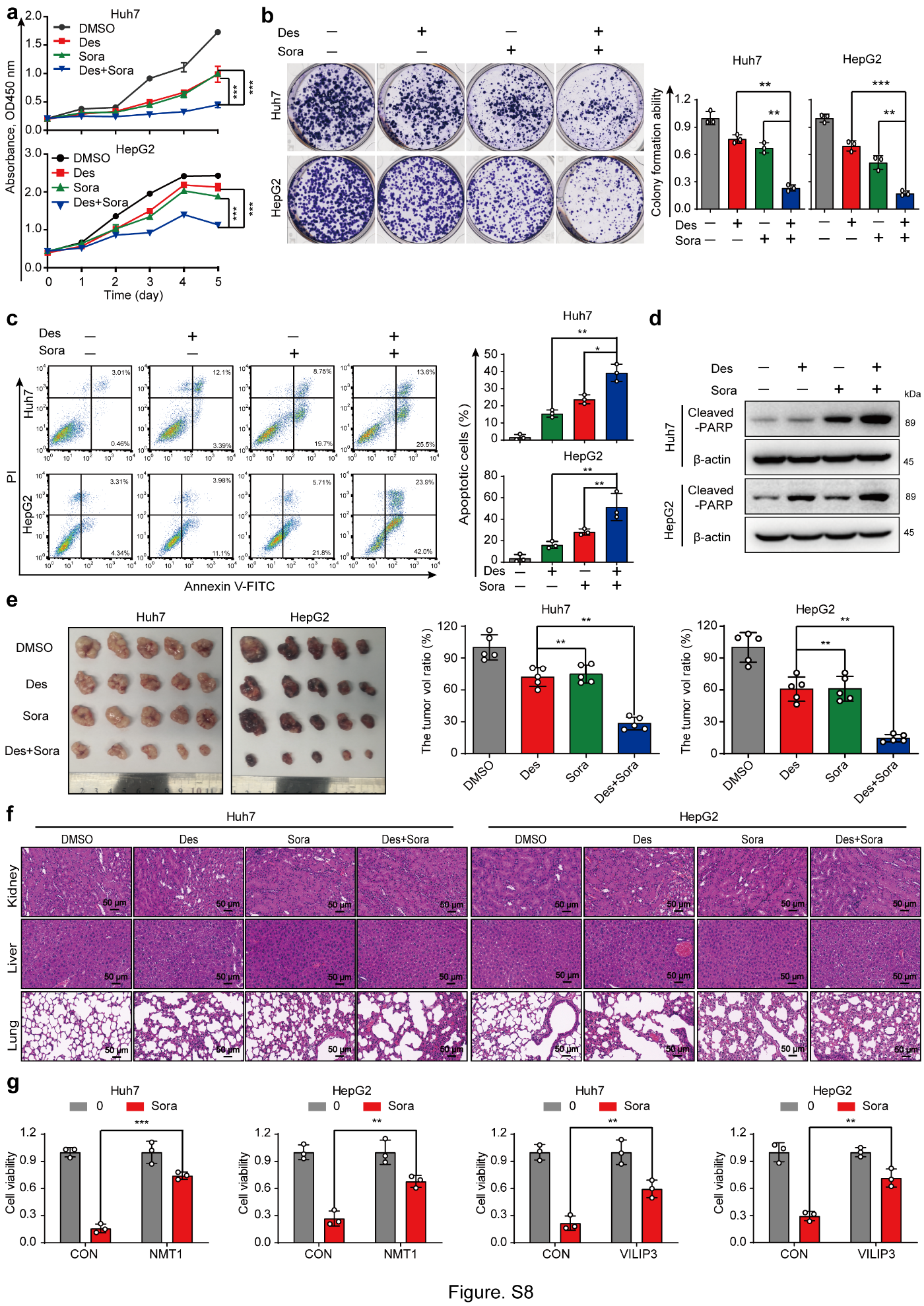


Figure. S8. Desloratadine sensitizes HCC cells to sorafenib treatment. a HCC cells were treated with 6 µg/ml desloratadine and 2 µg/ml sorafenib separately or in combination, and cell viability was evaluated by a CCK-8 assay. b HCC cells were treated with 4 µg/ml desloratadine and 2 µg/ml sorafenib separately or in combination, and the colony formation ability was evaluated. c HCC cells were treated with 8 µg/ml desloratadine and 8 µg/ml sorafenib separately or in combination, and apoptosis was analyzed by flow cytometry. d The level of cleaved PARP was analyzed by Western blot. β-actin served as the internal control. Nude mice bearing Huh7- or HepG2-derived xenografts were orally administered vehicle, desloratadine (15 mg/kg) or sorafenib (15 mg/kg) alone or a combination of desloratadine with sorafenib once every two days (n=5 mice per group). e Representative images of the tumors and tumor growth curves are shown. f Representative images of H&E staining in mouse organ tissue. g CCK-8 assays were performed to determine cell viability of NMT1 or VILIP3-overexpressing HCC cells treated with 4 μg/ml sorafenib for 48 h. Bars, SDs; *, p <0.05; **, p <0.01, ***, p <0.001.


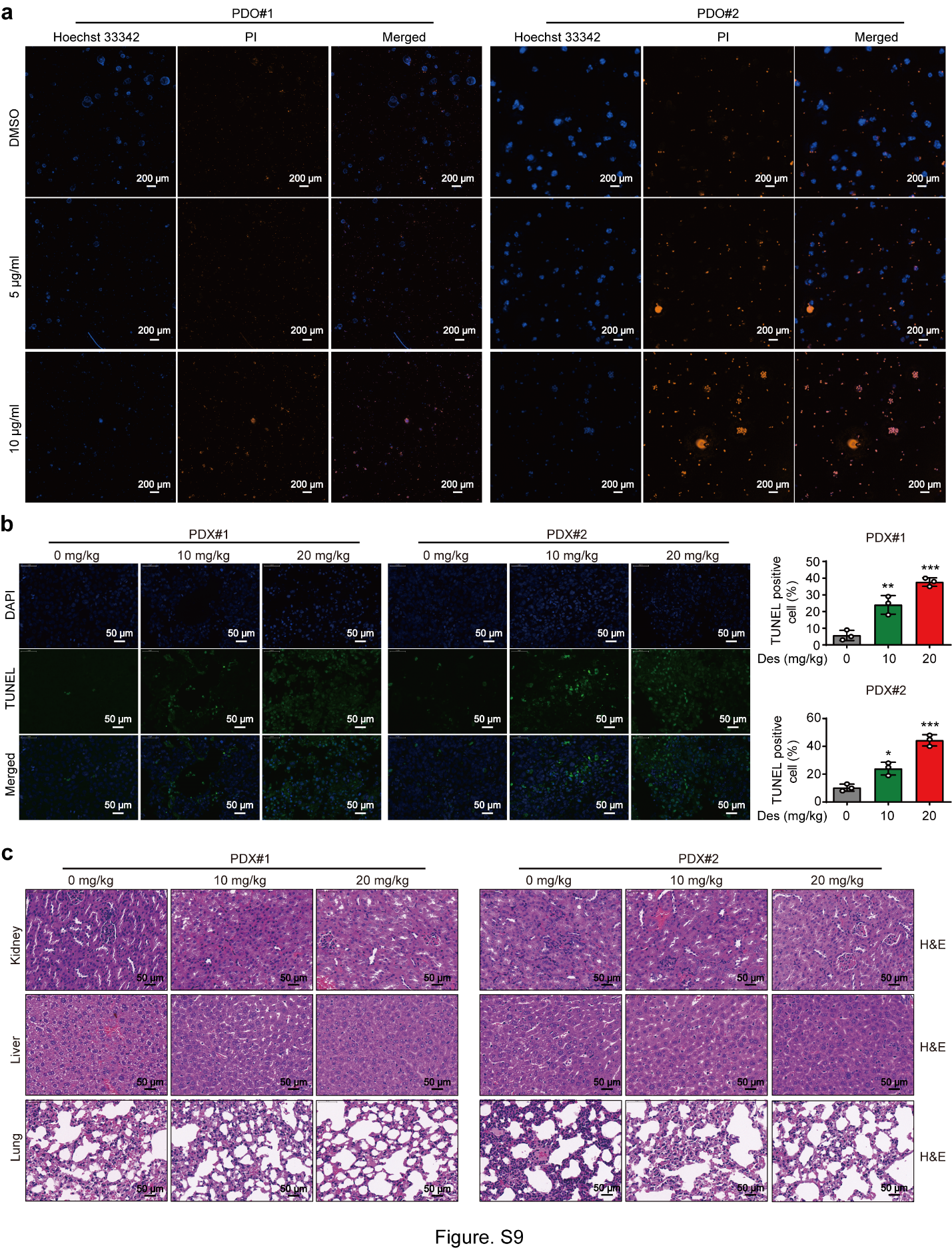


Figure. S9. Desloratadine suppresses HCC progression in PDO and PDX models. a PDO#1 and PDO#2 cells were treated with desloratadine (5 μg/ml or 10 μg/ml) or vehicle (DMSO) for 96 h, and morphological apoptosis analysis of PDO cells was performed by Hoechst 33342 and PI staining. b TUNEL assay results in the desloratadine-treated mice and the vehicle-treated mice (blue: DAPI, green: TUNEL). c Hematoxylin and eosin (H&E) staining of kidney, liver and lung sections from the PDX models. Bars, SDs; *, p <0.05, **, p <0.01, ***, p <0.001.

Table S1. The inhibition rate of 20 candidate compounds in HCC cells.

|  | Name | Inhibition rate (%) | | Is there any related  anti-tumor research |
| --- | --- | --- | --- | --- |
|  |  | Huh7 | HepG2 |  |
| 1 | Irbesartan | 71.8 | 68.5 | Yes |
| 2 | Orantinib | 71.8 | 96.9 | Yes |
| 3 | Mycophenolate Mofetil | 57.9 | 74.8 | Yes |
| 4 | Amiloride HCl | 76.5 | 69.6 | Yes |
| 5 | Enoxacin | 50.3 | 82.0 | Yes |
| 6 | Diltiazem HCl | 56.3 | 89.3 | Yes |
| 7 | Atracurium Besylate | 57.3 | 88.2 | Yes |
| 8 | Mestranol | 55.5 | 64.4 | Yes |
| 9 | Argatroban | 72.0 | 87.6 | Yes |
| 10 | Mubritinib | 50.0 | 72.9 | Yes |
| 11 | Licofelone | 56.0 | 69.3 | Yes |
| 12 | Dextrose | 68.0 | 57.6 | Yes |
| 13 | Neratinib | 85.2 | 81.6 | Yes |
| 14 | Erismodegib | 52.0 | 72.4 | Yes |
| 15 | L-carnitine | 54.1 | 89.4 | Yes |
| 16 | Desloratadine | 61.7 | 75.5 | No |
| 17 | Succinylsulfathiazole | 50.8 | 72.6 | No |
| 18 | Prochlorperazine | 97.1 | 83.3 | Yes |
| 19 | Nitroxoline | 73.1 | 73.4 | Yes |
| 20 | Fusidate Sodium | 53.5 | 76.8 | No |

Table S2. The list of 53 potential target proteins of desloratadine.

| **Number** | **Name** | NC-1 | NC-2 | NC-3 | Des-1 | Des-2 | Des-3 |
| --- | --- | --- | --- | --- | --- | --- | --- |
| 1 | DPOLA | Filtered | Filtered | Filtered | 17319 | 11053 | 6762 |
| 2 | SC24B | Filtered | Filtered | Filtered | 11728 | 22502 | 20411 |
| 3 | HS105 | Filtered | Filtered | Filtered | 34114 | 53384 | 22936 |
| 4 | UBP24 | Filtered | Filtered | Filtered | 980066 | 130656 | 510079 |
| 5 | OTUD5 | Filtered | Filtered | Filtered | 16904 | 65106 | 37347 |
| 6 | VDAC2 | Filtered | Filtered | Filtered | 3032 | 82286 | 8534 |
| 7 | IPO11 | Filtered | Filtered | Filtered | 31800 | 17712 | 23538 |
| 8 | BZW2 | Filtered | Filtered | Filtered | 30451 | 21030 | 20956 |
| 9 | NMT1 | Filtered | Filtered | Filtered | 18856 | 29852 | 27980 |
| 10 | MAGI3 | Filtered | Filtered | Filtered | 67185 | 69722 | 65410 |
| 11 | NAMPT | Filtered | Filtered | Filtered | 61175 | 55661 | 74177 |
| 12 | AHNK | Filtered | Filtered | Filtered | Filtered | 39503 | 34143 |
| 13 | PYR1 | Filtered | Filtered | Filtered | 54401 | Filtered | 59377 |
| 14 | DAAF5 | Filtered | Filtered | Filtered | 22700 | Filtered | 43354 |
| 15 | L1CAM | Filtered | Filtered | Filtered | 18177 | 24205 | Filtered |
| 16 | USP9X | Filtered | Filtered | Filtered | Filtered | 30634 | 52350 |
| 17 | NU160 | Filtered | Filtered | Filtered | 5698 | 17176 | Filtered |
| 18 | AMPB | Filtered | Filtered | Filtered | Filtered | 84474 | 44535 |
| 19 | STK4 | Filtered | Filtered | Filtered | 25861 | 50157 | Filtered |
| 20 | CLIC4 | Filtered | Filtered | Filtered | 1961 | 1633 | Filtered |
| 21 | PKHO1 | Filtered | Filtered | Filtered | 102835 | 79118 | Filtered |
| 22 | NSUN4 | Filtered | Filtered | Filtered | 45310 | 43252 | Filtered |
| 23 | ADT2 | Filtered | Filtered | Filtered | Filtered | 10358 | 5573 |
| 24 | TLN1 | Filtered | Filtered | Filtered | 2925 | 11811 | Filtered |
| 25 | PDIA6 | Filtered | Filtered | Filtered | 25437 | Filtered | 23772 |
| 26 | ANXA1 | Filtered | Filtered | Filtered | 715584 | Filtered | 492176 |
| 27 | CSN7A | Filtered | Filtered | Filtered | Filtered | 25215 | 25612 |
| 28 | PRKDC | Filtered | Filtered | Filtered | Filtered | 2250 | 21969 |
| 29 | DNJA1 | Filtered | Filtered | Filtered | 35324 | 10232 | Filtered |
| 30 | P5CS | Filtered | Filtered | Filtered | 6709 | 10635 | Filtered |
| 31 | RFC4 | Filtered | Filtered | Filtered | Filtered | 744171 | 613471 |
| 32 | IDI1 | Filtered | Filtered | Filtered | Filtered | 199606 | 97996 |
| 33 | MOCOS | Filtered | Filtered | Filtered | Filtered | 32975 | 51014 |
| 34 | MMS19 | Filtered | Filtered | Filtered | 1185 | 2716 | Filtered |
| 35 | HS90A | Filtered | Filtered | Filtered | 137063 | Filtered | 74637 |
| 36 | AMPL | Filtered | Filtered | Filtered | 57732 | 44264 | Filtered |
| 37 | AMPB | Filtered | Filtered | Filtered | 7278 | 26183 | Filtered |
| 38 | SYVC | Filtered | Filtered | Filtered | 61715 | Filtered | 14059 |
| 39 | CC154 | Filtered | Filtered | Filtered | 11987 | 17676 | Filtered |
| 40 | PRKDC | Filtered | Filtered | Filtered | 50055 | Filtered | 53430 |
| 41 | PNPH | Filtered | Filtered | Filtered | 56438 | Filtered | 838 |
| 42 | ABHEB | Filtered | Filtered | Filtered | Filtered | 8944 | 37297 |
| 43 | LMAN2 | Filtered | Filtered | Filtered | 12900 | 13950 | Filtered |
| 44 | PRI1 | Filtered | Filtered | Filtered | Filtered | 2453 | 1499 |
| 45 | RL4 | Filtered | Filtered | Filtered | 40941 | 83627 | Filtered |
| 46 | EIF3M | Filtered | Filtered | Filtered | 1238 | Filtered | 20535 |
| 47 | RT09 | Filtered | Filtered | Filtered | 6786 | Filtered | 19393 |
| 48 | SYQ | Filtered | Filtered | Filtered | 51608 | 46495 | Filtered |
| 49 | STT3B | Filtered | Filtered | Filtered | 55830 | 28164 | Filtered |
| 50 | SCOT1 | Filtered | Filtered | Filtered | 218045 | Filtered | 193832 |
| 51 | AGM1 | Filtered | Filtered | Filtered | Filtered | 6912 | 9955 |
| 52 | PUR2 | Filtered | Filtered | Filtered | Filtered | 1809 | 2220 |
| 53 | PRP8 | Filtered | Filtered | Filtered | 6304 | Filtered | 6698 |

Table S3. Correlation between NMT1 expression levels and clinic pathological parameters in 180 cases patients of HCC.

| **Variable** | ***n*** | **Low NMT1** | **High NMT1** | ***P* value** |
| --- | --- | --- | --- | --- |
| Age (years) |  |  |  |  |
| ≤55 | 104 | 55 | 49 |  |
| *>*55 | 76 | 39 | 37 | 0.835 |
|  |  |  |  |  |
| Gender |  |  |  |  |
| Male | 154 | 80 | 74 |  |
| Female | 26 | 14 | 12 | 0.858 |
|  |  |  |  |  |
| T-Stage |  |  |  |  |
| 1/2 | 132 | 76 | 55 |  |
| 3/4 | 48 | 18 | 31 | 0.011 |
|  |  |  |  |  |
| Grade |  |  |  |  |
| I & II | 119 | 70 | 49 |  |
| III & IV | 61 | 24 | 37 | 0.013 |
|  |  |  |  |  |
| Tumor size |  |  |  |  |
| ≤5 cm | 96 | 61 | 35 |  |
| *>*5 cm | 84 | 33 | 51 | 0.001 |

Table S4. The list of 21 potential substrate proteins for NMT1.

| **Number** | **Name** | **Proteins with N-terminal glycine** |
| --- | --- | --- |
| 1 | VILIP3 | MGKQNSKLR |
| 2 | PPM1G | MGAYLSQPN |
| 3 | FAM129A | MGGSASSQL |
| 4 | BRI3BP | MGARASGGP |
| 5 | PRKAB1 | MGNTSSERA |
| 6 | CKAP5 | MGDDSEWLK |
| 7 | IQGAP2 | MGCFKGVVA |
| 8 | AGL | MGHSKQIRI |
| 9 | PIK3R4 | MGNQLAGIA |
| 10 | ERCC5 | MGVQGLWKL |
| 11 | WDR3 | MGLTKQYLR |
| 12 | FYN | MGCVQCKDK |
| 13 | SAMM50 | MGTVHARSL |
| 14 | PPOX | MGRTVVVLG |
| 15 | GPR180 | MGGLRLLAV |
| 16 | EIPR1 | MGDGKKIIS |
| 17 | AGPAT3 | MGLLAFLKT |
| 18 | ACAT2 | MGSHPVLRI |
| 19 | RABGGTB | MGTPQKDVI |
| 20 | ZFPL1 | MGLCKCPKR |
| 21 | TTC1 | MGEKSENCG |

Table S5. Correlation between VILIP3 expression levels and clinic pathological parameters in 180 cases patients of HCC.

| **Variable** | ***n*** | **Low VILIP3** | **High VILIP3** | ***P* value** |
| --- | --- | --- | --- | --- |
| Age (years) |  |  |  |  |
| ≤55 | 104 | 34 | 70 |  |
| *>*55 | 76 | 35 | 41 | 0.058 |
|  |  |  |  |  |
| Gender |  |  |  |  |
| Male | 154 | 58 | 96 |  |
| Female | 26 | 11 | 15 | 0.652 |
|  |  |  |  |  |
| T-Stage |  |  |  |  |
| 1/2 | 132 | 58 | 74 |  |
| 3/4 | 48 | 11 | 37 | 0.010 |
|  |  |  |  |  |
| Grade |  |  |  |  |
| I & II | 115 | 51 | 64 |  |
| III & IV | 65 | 18 | 47 | 0.027 |
|  |  |  |  |  |
| Tumor size |  |  |  |  |
| ≤5 cm | 96 | 42 | 54 |  |
| *>*5 cm | 84 | 27 | 57 | 0.110 |

Table S6. The target sequences of shRNA, sgRNA and siRNA.

| **Name** | **Sequence** |
| --- | --- |
| shNMT1#1 | CCGGGGTACTTGAAGCAATTTCATTCTCGAGAATGAAATTGCTTCAAGTACCTTTTTG |
| shNMT1#2 | CCGGGGGCTGCGACCAATGGAAACTCGAGTTTCCATTGGTCGCAGCCCTTTTTG |
| sgVILIP3#1 | TCCTTGGAAGAATTCATCAG |
| sgVILIP3#2 | GTCTGTGATGAAGATGCCGG |
| siNMT1#1 | GGTACTTGAAGCAATTTCATT |
| siNMT1#2 | GGGCTGCGACCAATGGAAATT |
| siNMT2#1 | GCACATTATTGACACGTTTTT |
| siNMT2#2 | CATAAGAAGTTGAGATCGATT |
| siOUTD5#1 | AAAAATTCAACATTGAAGGCTTT |
| siOUTD5#2 | TTTAATAAAGAAAAAATTCAATT |
| siUSP9X#1 | GTCGTTACAGCTAGTATTT |
| siUSP9X#2 | CTGTGATTCAGCAACTCTATT |
| siNAMPT#1 | GGGTTGCAGTACATTCTTATT |
| siNAMPT#2 | CAAGGTTACTCACTATAAATT |
| siPRKDC#1 | GCTGGAACTACTCGTATAATT |
| siPRKDC#2 | CCGTTAGTCCTACCTTGAATT |

Table S7. The list of indicated antibodies.

| **Antibody** | **Cat No.** | **Manufacturer** |
| --- | --- | --- |
| Cleaved-PARP | 5625T | Cell Signaling Technology |
| Cyclin B1 | 12231T | Cell Signaling Technology |
| Ki67 | 9449T | Cell Signaling Technology |
| NMT1 | ab223729 | Abcam |
| NMT1 | sc-365723 | Santa Cruz Biotechnology |
| NMT2 | bs-6233R | Bioss Antibodies |
| p-Akt | 4060S | Cell Signaling Technology |
| Akt | 4685S | Cell Signaling Technology |
| p-JNK | 9255S | Cell Signaling Technology |
| p-p38 | 4511S | Cell Signaling Technology |
| p38 | 8069S | Cell Signaling Technology |
| p-ERK1/2 | 4370T | Cell Signaling Technology |
| Cleaved caspase-3 | 9661S | Cell Signaling Technology |
| VILIP3 | 10989-1-AP | Proteintech |
| p-p65 | GB11142-1 | Servicebio |
| P65 | GB11142 | Servicebio |
| Bcl-2 | 60178-lg | Proteintech |
| FAM129A | 21333-1-AP | Proteintech |
| PPM1G | 15532-1-AP | Proteintech |
| β-actin | 4970S | Cell Signaling Technology |

Table S8. The list of primers used for qRT-PCR or PCR.

| **Name** | | | **Primer Sequence** |  |
| --- | --- | --- | --- | --- |
| NMT1 | Forward | 5’-AGTGAGACAGCAGTGAAGCC-3’ | | |
|  | Reverse | 5’-CTCATTCTCGCAATCGCTGC-3’ | | |
| OUTD5 | Forward | 5’-GGTTGTGCGAAAGCATTGCAT-3’ | | |
|  | Reverse | 5’-ACCTCCACAGGACGGTTGT-3’ | | |
| USP9X | Forward | 5’-TCGGAGGGAATGACAACCAG-3’ | | |
|  | Reverse | 5’-GGAGTTGCCGGGGAATTTTCA-3’ | | |
| NAMPT | Forward | 5’-AATGTTCTCTTCACGGTGGAAAA-3’ | | |
|  | Reverse | 5’-ACTGTGATTGGATACCAGGACT-3’ | | |
| PRKDC | Forward | 5’-AGCTGGCTTGCGCCTATTT-3’ | | |
|  | Reverse | 5’-GGGCACACCACTTTAACAAGAC-3’ | | |
| VILIP3 | Forward | 5’-GAGAACACGGAGTTCACCGAC-3’ | | |
|  | Reverse | 5’-ACAGTTTGCCGTCATTGTTGG-3’ | | |
| Bcl-2 | Forward | 5’- GGTGGGGTCATGTGTGTGG-3’ | | |
|  | Reverse | 5’- CGGTTCAGGTACTCAGTCATCC-3’ | | |
| NMT1^P204A^ | Forward | 5’-TCTCCGGCCAgCCGGCTGGCT-3’ | | |
|  | Reverse | 5’-GCCCACAAAAGAAACTCCGGGGAATAATCAAATCGG-3’ | | |
| NMT1^N246A^ | Forward | 5’-GGTAGAGATCgcCTTCCTGTGTGTC-3’ | | |
|  | Reverse | 5’-ATCTTCTTCTCTGTGTCATAG-3’ | | |
| NMT1^W374A^ | Forward | 5’-GGTGGAGCACgcGTTCTACCCCC-3’ | | |
|  | Reverse | 5’-TCCTCCTGGCTCATGACG-3’ | | |
| NMT1^Q496A^ | Forward | 5’-ACTGGTGCTAgcAGGATCCGGATCCG-3’ | | |
|  | Reverse | 5’-CCAACCTTCTCTGCCCCC-3’ | | |
| VILIP3^mut^ | Forward  Reverse | 5’-GCCACCATGGcCAAACAGAAC-3’  5’-GAATTCGCTAGCTCTAGAATC-3’ | | |

Data S1. (separate file)

**The list of 419 FDA-approved compound library.**
